# A metabologenomics approach to unlock the metabolome of the novel Antarctic deep-sea isolate *Lacinutrix shetlandiensis* sp. nov. WUR7

**DOI:** 10.1093/pnasnexus/pgad221

**Published:** 2023-07-06

**Authors:** Giovanni A Vitale, Grant G January, Ernest Oppong-Danquah, Gerardo Della Sala, Fortunato Palma Esposito, Deniz Tasdemir, Donatella de Pascale

**Affiliations:** CMFI Cluster of Excellence, Interfaculty Institute of Microbiology and Medicine, University of Tübingen, Tübingen 72076, Germany; Department of Ecosustainable Marine Biotechnology, Stazione Zoologica Anton Dohrn, Giardini Molosiglio, Via F.A. Acton 55, 80133, Naples, Italy; Institute of Biochemistry and Cell Biology (IBBC), National Research Council, Via Pietro Castellino 111, 80131 Naples, Italy; Derriford Research Facility, School of Biomedical Sciences, Faculty of Health, University of Plymouth, 14 Research Way, Plymouth PL6 8BU, UK; Institute of Biochemistry and Cell Biology (IBBC), National Research Council, Via Pietro Castellino 111, 80131 Naples, Italy; GEOMAR Centre for Marine Biotechnology (GEOMAR-Biotech), Research Unit Marine Natural Products Chemistry, GEOMAR Helmholtz Centre for Ocean Research Kiel, Am Kiel-Kanal 44, Kiel 24106, Germany; Department of Ecosustainable Marine Biotechnology, Stazione Zoologica Anton Dohrn, Giardini Molosiglio, Via F.A. Acton 55, 80133, Naples, Italy; Department of Ecosustainable Marine Biotechnology, Stazione Zoologica Anton Dohrn, Giardini Molosiglio, Via F.A. Acton 55, 80133, Naples, Italy; Institute of Biochemistry and Cell Biology (IBBC), National Research Council, Via Pietro Castellino 111, 80131 Naples, Italy; GEOMAR Centre for Marine Biotechnology (GEOMAR-Biotech), Research Unit Marine Natural Products Chemistry, GEOMAR Helmholtz Centre for Ocean Research Kiel, Am Kiel-Kanal 44, Kiel 24106, Germany; Kiel University, Christian-Albrechts-Platz 4, Kiel 24118, Germany; Department of Ecosustainable Marine Biotechnology, Stazione Zoologica Anton Dohrn, Giardini Molosiglio, Via F.A. Acton 55, 80133, Naples, Italy; Institute of Biochemistry and Cell Biology (IBBC), National Research Council, Via Pietro Castellino 111, 80131 Naples, Italy

**Keywords:** deep sea, *Lacinutrix*, OMICs, OSMAC, alkaloids

## Abstract

The South Shetland Trough, Antarctica, is an underexplored region for microbiological and biotechnological exploitation. Herein, we describe the isolation and characterization of the novel bacterium *Lacinutrix shetlandiensis* sp. nov. WUR7 from a deep-sea environment. We explored its chemical diversity via a metabologenomics approach, wherein the OSMAC strategy was strategically employed to upregulate cryptic genes for secondary metabolite production. Based on hybrid de novo whole genome sequencing and digital DNA–DNA hybridization, isolate WUR7 was identified as a novel species from the Gram-negative genus *Lacinutrix*. Its genome was mined for the presence of biosynthetic gene clusters with limited results. However, extensive investigation of its metabolism uncovered an unusual tryptophan decarboxylase with high sequence homology and conserved structure of the active site as compared to ZP_02040762, a highly specific tryptophan decarboxylase from *Ruminococcus gnavus*. Therefore, WUR7's metabolism was directed toward indole-based alkaloid biosynthesis by feeding it with *L*-tryptophan. As expected, its metabolome profile changed dramatically, by triggering the extracellular accumulation of a massive array of metabolites unexpressed in the absence of tryptophan. Untargeted LC-MS/MS coupled with molecular networking, followed along with chemoinformatic dereplication, allowed for the annotation of 10 indole alkaloids, belonging to β-carboline, bisindole, and monoindole classes, alongside several unknown alkaloids. These findings guided us to the isolation of a new natural bisindole alkaloid 8,9-dihydrocoscinamide B (**1**), as the first alkaloid from the genus *Lacinutrix*, whose structure was elucidated on the basis of extensive 1D and 2D NMR and HR-ESIMS experiments. This comprehensive strategy allowed us to unlock the previously unexploited metabolome of *L. shetlandiensis* sp. nov. WUR7.

**Significance Statement**

Microorganisms are an invaluable source of secondary metabolites with renowned therapeutic potential, such as alkaloids. However, most of them remain inaccessible under standard laboratory conditions, majorly due to the lack of the right stimulus to boost their production. Finding the right conditions is challenging but represents the key to unlock a treasure trove of secondary metabolites. Herein, the new deep-sea Antarctic strain *Lacinutrix shetlandiensis* sp. nov. WUR7 was isolated. Genomics and protein modeling suggested culture condition refinement and *L*-tryptophan supplementation, which triggered the production of a large number of alkaloids, as unveiled through metabolomics and chemoinformatics. Purification and full spectroscopic analyses led to the characterization of 8,9-dihydrocoscinamide B, herein being reported for the first time from a natural source.

## Introduction

Environmental stressors on marine micro- and macroorganisms have led to unique evolution systems and adaptation strategies, triggering the development of unique metabolic pathways, culminating in the production of secondary metabolites (SMs). The deep-sea cold trenches of Antarctica still represent a vastly underexplored ecosystem; in particular, the South Shetland Trough (SST) is considered a polyextreme environment as it lies near the Antarctic Peninsula and is one of the few cryogenic deep-sea trenches in the world. Despite its uniqueness, which potentially makes it an ideal candidate for the isolation of talented microorganisms, its impervious conditions had limited its exploration. Although the isolation of new or potentially prolific strains is a complex task, the rediscovery of known molecules represents the main bottleneck in natural product (NP) research (1). In this context, recent advances in “OMIC” technologies including genomics and metabolomics are drastically leading us into a “new Golden Age of NPs.” Metabolomics is currently making large strides with respect to dereplication which is largely attributable to the rise of cheminformatic tools including Molecular Networking implemented on the Global Natural Product Social Molecular Networking (GNPS) (2) platform, alongside numerous databases and tools for chemical class and structure prediction (3). Concomitantly, numerous bioinformatic tools for genome annotation and detection of biosynthetic gene clusters (BGCs), including antiSMASH (4) and BlastKOALA (5), can be used to predict the class and sometimes the structure of encoded metabolites. Genome mining demonstrated that the number of BGCs encoding for SMs is larger than the number of SMs effectively produced, but most of them often remain inaccessible under “standard laboratory conditions.” Moreover, only a small portion of in silico–predicted BGCs have been characterized in vitro (6). A practical approach to overcome SM inaccessibility is the “One Strain Many Compounds” (OSMAC) approach; its basic principle is that by providing different environmental stimuli, including nutrients, physical parameters, chemical elicitors, and microbial cocultivation, silent or cryptic BGCs can be activated, giving access to otherwise inaccessible SMs (6–9). In this study, a comprehensive metabologenomics approach was employed to investigate the potential of a novel strain belonging to the genus *Lacinutrix*, isolated from deep-sea sediments originating from the SST. The rare genus *Lacinutrix*, which belongs to the *Flavobacteriaceae* family, consists of 12 bacterial species, isolated mostly from marine organisms and sediments (10), with this genus remaining rather unexplored for its secondary metabolism. Thus far, only two isobranched lyso-ornithine lipids have been reported from an Artic *Lacinutrix* species (11). Therefore, this study delved into exploring the genome and metabolome of *Lacinutrix shetlandiensis* sp. nov. WUR7 (henceforth referred to as simply “WUR7”) aiming to enrich the NP pool.

Herein, the genome of WUR7 was sequenced, annotated and analyzed with antiSMASH 5.0 (4). However, a preliminary genome annotation of BGCs did not uncover intriguing features, neither did the preliminary metabolic profiling in its isolation medium. Nonetheless, extensive investigation of its metabolic pathways revealed the unexpected presence of a tryptophan decarboxylase (TDC), i.e. WP_203458448, an enzyme involved in the biosynthesis of tryptamine, commonly encountered in the plant kingdom, where it is involved in alkaloid biosynthesis but extraordinarily rare in bacteria (12). To date, TDCs have been recently observed in a low percentage of human gut microbiota where tryptamine acts as a neurotransmitter (13), while there are no reports of TDCs from Antarctic bacteria. Nevertheless, recent findings disclosed Tryptophan (Trp) decarboxylation as a fundamental step in the biosynthesis of certain bacterial alkaloids (14–16).

Comparative genomics and protein modeling revealed that WP_203458448 shares a high identity, with respect to the active sites, with ZP_02040762, a decarboxylase with high selectivity for Trp, recently described from *Ruminococcus gnavus* (13).

Based on these findings, we hypothesized to modulate WUR7's metabolism by supplementing *L*-Trp into its culture medium, in hopes that we could access its unexpressed metabolites. This hypothesis was substantiated as the supplantation of Trp resulted in a marked alteration of WUR7's metabolic profile. Thereafter, untargeted Liquid Chromatography-Mass Spectrometry/Mass Spectrometry (LC-HRMS/MS) followed by molecular networking analysis and chemoinformatic classification of the molecular clusters revealed the production of indole alkaloids (17) constituting the vast majority of WUR7's metabolome (35% of nodes) and enabled the annotation of putatively known alkaloids along with many other new entities. Extensive purification of a WUR7 organic extract led to the isolation and structure elucidation of 8,9-dihydrocoscinamide B, herein, being reported for the first time as a NP and showing antimicrobial activity against *Staphylococcus aureus* and methicillin-resistant *S. aureus* (MRSA). This combined approach allowed us to uncover a hidden alkaloid factory from the genus *Lacinutrix*.

## Results

WUR7 was isolated from deep-sea sediments originating from the SST, which were collected in the framework of the Eurofleet 14-010 call 2013 project entitled PharmaDEEP. By using a hybrid sequencing approach consisting of Pacific Bioscience's (PacBio) single-molecule real-time sequencing (SMRT), followed by Illumina NextSeq 500 sequencing for error correction of the de novo–assembled genome, we were able to assemble the entire circular genome of WUR7 (125× coverage). To date, there are 21 genomes from *Lacinutrix* strains present in NCBI GenBank (18), with only five of them being deposited as full genomes (for genome statistics and visualization of the circularized genome, see Tables 1 and S1 and Fig. S1).

**Table 1.** Genome attributes of *L. shetlandiensis* sp. nov. WUR7.

	Number	% of total
**DNA, total number of bases**	3,987,475	100.00%
DNA coding number of bases	3,600,562	90.30%
DNA G + C number of bases	1,296,092	32.50%
**DNA scaffolds**	1	100.00%
**Genes total number**	3,559	100.00%
Protein coding genes	3,449	96.91%
Regulatory and miscellaneous features	52	1.46%
RNA genes	58	1.63%
rRNA genes	12	0.34%
tRNA genes	43	1.21%
Other RNA genes	3	0.08%

Main features are indicated in boldface.

To taxonomically delineate WUR7, pairwise comparisons were conducted using the Type Strain Genome Server (TYGS), which showed 50.4% digital DNA–DNA hybridization (dDDH) to *Lacinutrix himadriensis* E4-9a(T) (Tables 2 and S2). The threshold for correct taxonomic assignment using dDDH is ε 70% for species classification, and therefore, based on these results and whole genome phylogeny, we have assigned this isolate as a new species, for which we propose the name *L. shetlandiensis* sp. nov. WUR7. *L. shetlandiensis* sp. nov. WUR7 is included within the same species clade as the type strain *L*. *himadriensis* E4-9a(T) (Fig. S2).

**Table 2.** Pairwise comparisons of WUR7 genome vs. related type strain genomes at the genus level by using the online TYGS^a^

Subject strain	dDDH (d0, %)	C.I. (d0, %)	dDDH (d4, %)	C.I. (d4, %)	dDDH (d6, %)	C.I. (d6, %)	G +C content difference (%)
***L. himadriensis* E4-9a**	69.5	(65.6–73.1)	**50**.**4**	**(47.8–53.0)**	67.1	(63.7–70.3)	0.1
*Lacinutrix algicola* AKS293	18.4	(15.3–22.0)	22.6	(20.3–25.1)	18.2	(15.5–21.2)	1.14
*Lacinutrix mariniflava* AKS432	17.6	(14.5–21.1)	22.2	(20.0–24.7)	17.4	(14.8–20.4)	0.73
*Lacinutrix jangbogonensis* PAMC 27137	16	(13.0–19.4)	20.8	(18.5–23.2)	16	(13.4–18.9)	0.32
*Lacinutrix venerupis* DSM 28755	17	(14.0–20.5)	20	(17.8–22.5)	16.8	(14.2–19.7)	2.06

The name of the closest strain to WUR7 is highlighted in bold.

C.I., confidence interval; *d0*, *d4*, and *d6* refer to different algorithms used in TYGS analysis.

a These data disclosed *L. himadriensis* E4-9a(T) as the closest type strain related to *L. shetlandiensis* sp. nov. WUR7. The probability that this value is correct was confirmed by the confidence interval that was between 47.8% and 53.0% by linear regression.

Bioinformatic analysis of WUR7's genome using antiSMASH predicted the presence of three putative BGCs encoding for the biosynthesis of an aryl polyene-resorcinol, a terpene, and a ladderane (Table S3). In addition, WUR7's genome was annotated through BlastKOALA to search for biochemical pathways and/or biocatalysts involved in SM biosynthesis, which may possibly have been missed during the antiSMASH analysis.

Notably, it evidenced the presence of a TDC (WP_203458448), an enzyme which is exceptionally rare in bacteria. Decarboxylation of Trp leads to the formation of tryptamine (Fig. S3), i.e. the starting building block for the biosynthesis of indole and β-carboline alkaloids, such as harmala derivatives (19). In silico screening of WUR7's genome shed light on the presence of an additional enzyme, i.e. WP_203460975, belonging to the amino acid decarboxylase class.

**Fig. 1.**
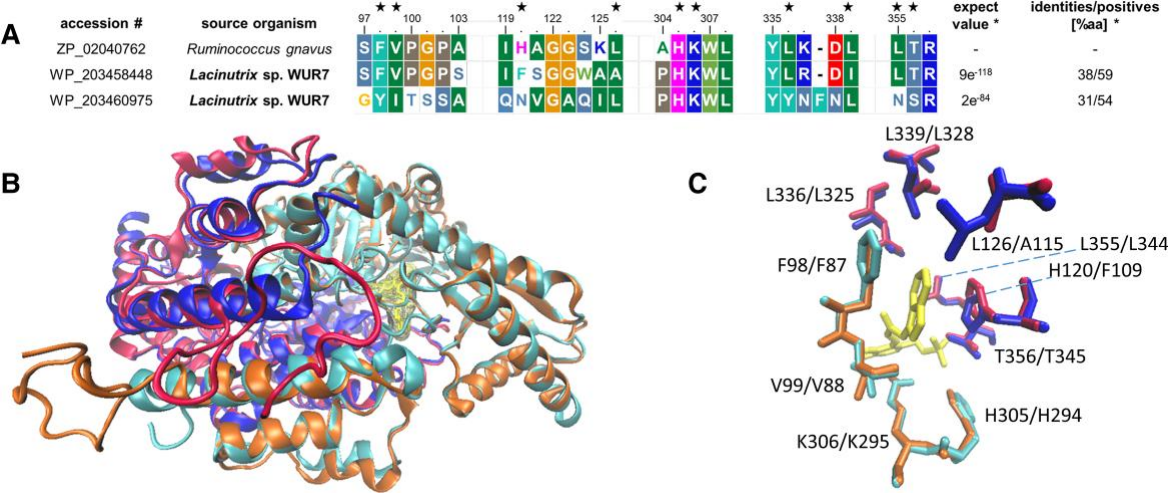
WUR7 decarboxylases WUR7_ADC1 and WUR7_ADC2 were aligned with the TDC ZP_02040762 from *R. gnavus*, to highlight key residues conferring Trp specificity according to the model proposed by Williams et al (11) which are indicated by the black star over the residue. A) Fragments of WUR7_ADC1 share high similarity (approximately 70% identity) with ZP_02040762. Proteins are numbered according to ZP_02040762. *Significance of BlastP alignments using ZP_02040762 as query and WUR7_ADC1 and WUR7_ADC2 as subjects; values are calculated over the entire amino acid sequence. B) Overall superimposition of WUR7_ADC1 (red and orange chains) on chains B (blue) and D (cyan) of ZP_02040762 crystal. C) 3D positioning of superimposed residues directly interacting with the indole-based inhibitor PLP-(S)-alpha-FMT ketone (yellow).

A BlastP search against the RCSB Protein Data Bank (RCSB PDB), WP_203458448 (WUR7_ADC1), and WP_203460975 (WUR7_ADC2) found ZP_02040762 as the closest homolog, which is a PLP-dependent decarboxylase from *R. gnavus*, displaying higher efficiency for Trp over phenylalanine (Phe) (>1,000 times higher catalytic efficiency). Although WUR7_ADC1 shares 38% homology with ZP_02040762 over the entire amino acid sequence, it presents quasi-identical structural components for selective binding of Trp (Fig. 1A). Furthermore, superimposition of the best model built for WUR7_ADC1 on ZP_02040762 (Fig. 1B), featuring a G factor average of −0.02 and 92.1% of residues in the most favored region (Fig. S4), revealed a nearly identical 3D disposition of residues conferring Trp selectivity (Fig. 1C). In light of these findings, WUR7_ADC1 has been predicted to bear high Trp specificity.

**Fig. 2.**
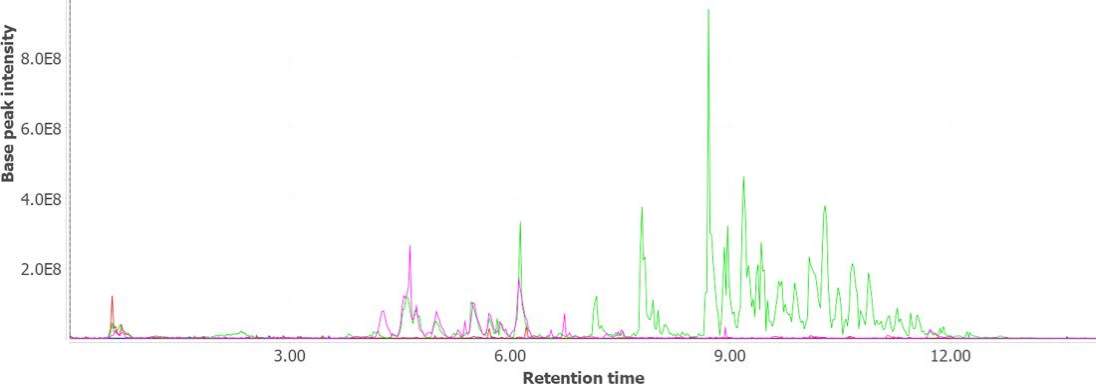
Overlapped visualization of the base peak chromatograms of the extracellular extracts obtained in MB (red profile), MB + tryptone (purple profile), and MB + tryptone + *L*-Trp (green profile), conditions 1, 2, and 3, respectively.

**Fig. 3.**
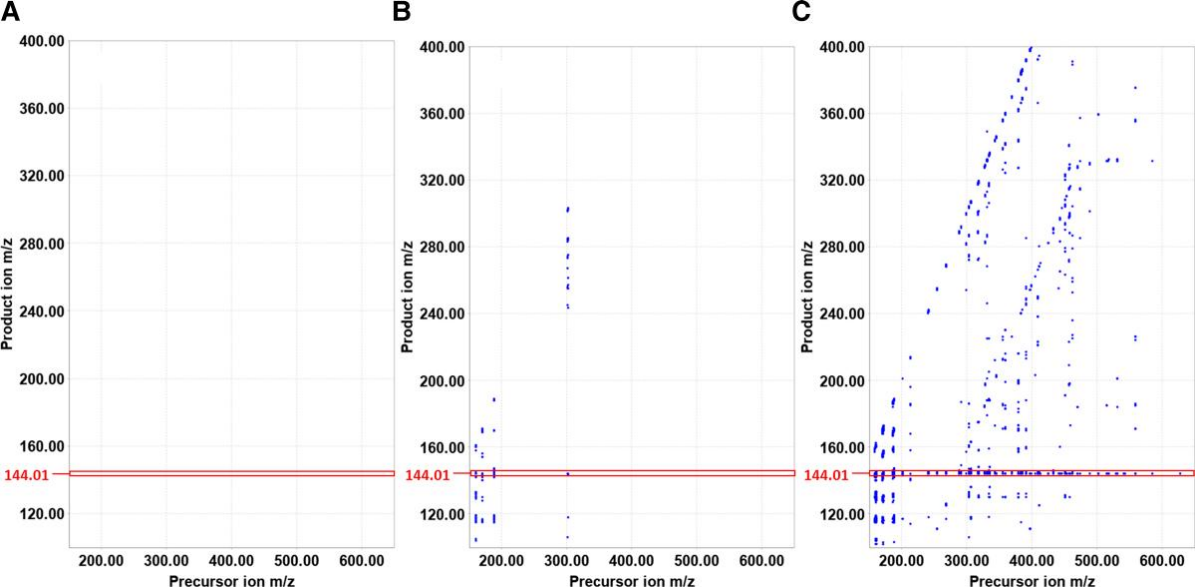
Comparative metabolic profiling of extracellular extracts from *L. shetlandiensis* sp. nov. WUR7 grown in culture conditions 1 A), 2 B), and 3 C). Product ion/precursor ion plots from MS/MS data of the extracellular extracts highlight precursor ions sharing the fragment ion at *m/z* 144.01 (red square). Tryptophan addition (condition 3C) triggers the secretion of a massive number of bacterial metabolites displaying a common structural motif, which has been later shown to be diagnostic of indole-based alkaloids.

The increased availability of an amine precursor is known to be one of the crucial steps to trigger alkaloid biosynthesis (12), and WUR7_ADC1 was supposed to be a regulatory enzyme of this pathway in the native host. Therefore, we hypothesized to feed WUR7 with *L*-Trp in order to elicit tryptamine accumulation by Trp decarboxylation, thereby enhancing the metabolic flux through biosynthetic routes leading to indole-based alkaloids. To monitor changes in its metabolome in response to this stimulus, WUR7 was cultivated in three conditions: (i) Marine Broth (MB) (isolation medium), (ii) MB + tryptone, and (iii) MB + tryptone + *L*-Trp. After 5 days of incubation, the supernatants were extracted as described in Materials and methods. Comparative metabolomic profiling of extracellular extracts 1–3 analyzed by UPLC-MS pointed to the presence of a massive number of metabolites expressed under condition 3, which were absent in conditions 1 and 2 (Fig. 2). Investigation of MS/MS data of the exclusive array of compounds revealed a product ion at *m/z* 144.01 as a mutual fragment, suggesting a common structural motif (Fig. 3A–C).

Therefore, an upscaled cultivation was prepared in condition 3 and the supernatant was extracted as described in Materials and methods. The extracellular extract was subjected to C18 solid-phase extraction (SPE) fractionation by using a H_2_O/MeOH gradient.

The molecular composition of the SPE fractions was investigated by untargeted Ultra Performance Liquid Chromatography (UPLC) coupled to High Resolution Mass Spectrometry (HRMS) Data Dependent Analysis (DDA), and molecular networking, in an attempt to holistically examine WUR7's metabolome. As previously reported (20), SPE fractions were employed for untargeted fragmentation data acquisition in order to decrease compound coelution during UHPLC separation and reduce coalescence events, thus improving quality/purity of product ion scans, for a more comprehensive overview of WUR7's metabolome.

**Fig. 4.**
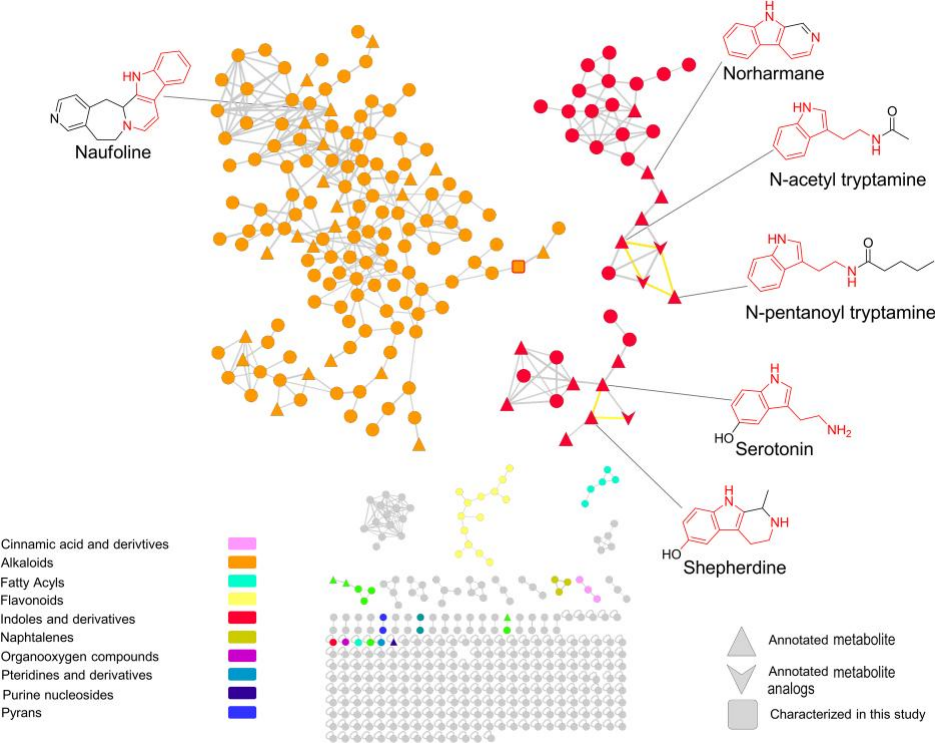
WUR7 extracellular extracts’ Molecular Network. The predicted molecular classes are identified by a specific color displayed in the color chart. The shape of the node indicates whether the compound was annotated by a combination of GNPS and The Dictionary of Natural Products (DNP) (triangle), or unannotated (circle). Only the structure of IDs consolidated by manual dereplication of the MS/MS spectra was reported. Tryptamine-derived moieties coming from Trp decarboxylation are drawn in red. Analogs of the annotated metabolites directly connected were also annotated (down arrow), and edges between them and annotated nodes are reported in yellow. The red-squared node in the alkaloid cluster corresponds to 8,9-dihydrocoscinamide B, purified and fully characterized in the present study.

The Feature-Based Molecular Network (FBMN) obtained was reanalyzed through the MolNetEnhancer workflow. Here, information deriving from in silico structure annotations from GNPS Library Search and Dereplicator was integrated into the network and chemical class assignment was performed using the ClassyFire algorithm. This process yielded a large molecular network, in which 12 different molecular classes were putatively assigned (Fig. 4). Remarkably out of 509 nodes, 140 were annotated as alkaloids and 39 as indole derivatives. Among them, 39 compounds found putative matches with indole alkaloids on the GNPS and Dictionary of Natural Products 31.1 (DNP) databases. Thereafter, experimental fragmentation spectra of these compounds were carefully examined to validate GNPS’s library matches (Figs. 4 and S5–S8 and Table S4).

**Fig. 5.**
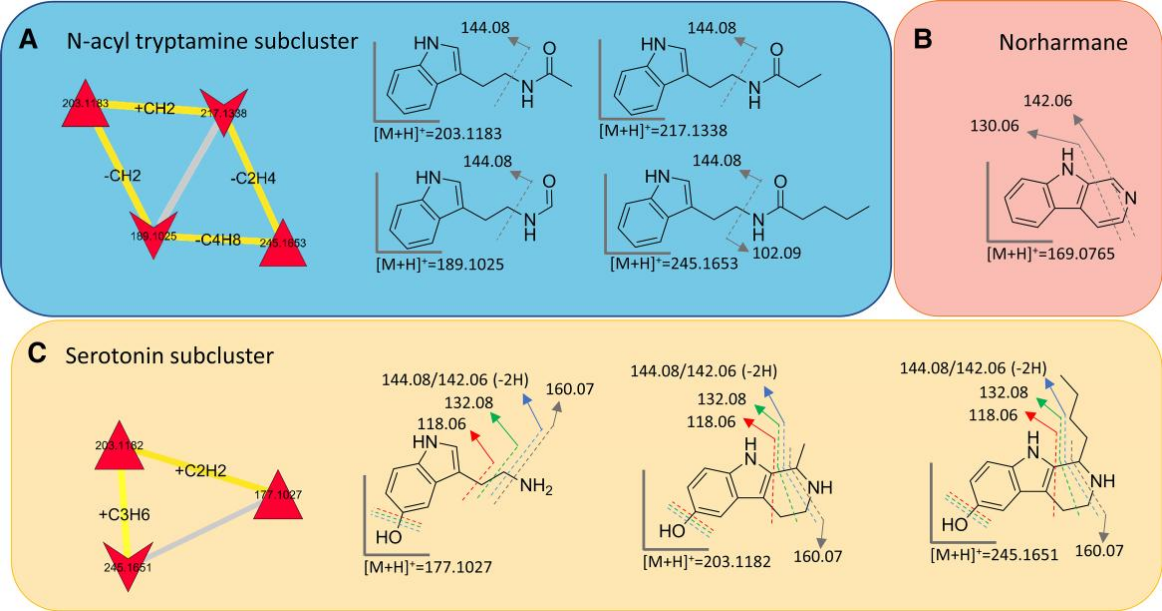
Molecular subclusters and structural annotations. Gray lines indicate single fragmentation, while colored lines indicate multiple fragmentations. A) *N*-acyl tryptamine subcluster was built around *N*-acetyl tryptamine and *N*-pentanoyl tryptamine nodes, which allowed the annotation of other two congeners. B) Typical fragmentations of norharmane detected in WUR7 extract. C) In the serotonin subcluster, two β-carbolines were putatively identified as shepherdine and a shepherdine novel homolog with a longer side chain.

Manual inspection of mass tandem spectra from alkaloids and indole derivatives consolidated the chemoinformatic prediction, as all these metabolites shared the fragment at *m/z* 144.0811 (C_10_H_10_N^+^). This is indicative of indole-based alkaloids, and it is due to the 3-ethyl-1H-indole fragment (21–24). Detection of *N*-acetyl tryptamine and *N*-pentanoyl tryptamine moved our attention to the closest neighbors in the molecular network, thus allowing for the annotation of two congeners featuring different *N*-acyl substituents, i.e. *N*-formyl and *N*-propanoyl tryptamines (Figs. 5A and S5). *N*-Acyl tryptamines are known to be precursors of β-carbolines in plants. Therefore, it was somehow not surprising to find β-carboline derivatives in the WUR7 extract, such as norharmane (Fig. 5B), which is supposed to come from *N*-formyl tryptamine according to the biosynthetic route described in plants (12). The structure of norharmane was confirmed as the experimental MS/MS spectrum matched perfectly with the one deposited in the GNPS reference spectral library (Table S4 and Fig. S6). The molecular network unveiled the presence of a serotonin subcluster (Fig. 5C), including shepherdine and a putative novel homolog, both bearing a 5-hydroxy tetrahydro β-carboline scaffold and arguably coming from serotonin. Shepherdine was identified as displaying complete identity with previous reported ESI–MS tandem spectrum (24). Sharing high similarity with the shepherdine fragmentation pathway, the structure of its homolog (*m/z* 245) was predicted and shown to bear a pendant butyl moiety instead of the methyl group present in shepherdine. Indeed, the [M+H]^+^ pseudomolecular ion of the 245 Da-homolog undergoes sequential losses of NH_3_ and C_5_H_8_, differently from shepherdine whose MS/MS spectrum shows a [M+H-NH_3_-C_2_H_2_]^+^ fragment ion (Figs. 5C and S7).

Numerous metabolites in the alkaloid cluster showed sequential neutral losses corresponding to 3-ethyl-1H-indole fragment, indicating the putative presence of bis- and tris-indole derivatives. The presence of the fragment 144.0452 (C_9_H_6_NO^+^) was another common feature retrieved in numerous unannotated nodes, along with the common 3-ethyl-1H-indole fragment, suggesting the presence of bis-indole alkaloids, but containing a 1H-Indole-3-carbonyl moiety.

Considering the lack of characterized SMs from the genus *Lacinutrix*, these findings encouraged us to investigate the fractions containing alkaloids and indole derivatives.

Based on the LC-HRMS profiles, the SPE fractions F75% and F100% were found to be the richest in alkaloids. F75% was selected for HPLC purification as showing rather less complex chemical profile compared to F100%.

**Fig. 6.**
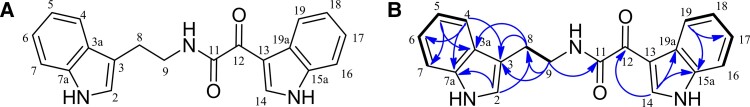
Structure of 8,9-dihydrocoscinamide B (**1**). A) Structure, and B) key COSY correlations in bold and HMBC highlighted with blue arrows.

F75% was subjected to two steps of reversed-phase HPLC separations, thus affording a fraction containing the pure compound **1**. Based on its HR-ESIMS data (*m/z* 332.1394 [M+H]^+^), the molecular formula was identified as C_20_H_18_N_3_O_2_^+^, indicating 14 degrees of unsaturation (DoUs). The ^1^H NMR spectrum (DMSO-*d*_6_) revealed ten olefinic methines (*δ*_H_ 6.98–8.75), two methylenes (*δ*_H_ 2.95 and 3.52), and three exchangeable protons (*δ*_H_ 8.81, 10.82, and 12.22) (Table S5). The ^13^C NMR data (Table S5) contained 19 carbon signals, which included two carbonyls (*δ*_C_ 163.5 and 182.2), two methylenes (*δ*_C_ 24.9 and 39.4), and 15 olefinic carbons (*δ*_C_ 111.4–138.5). The 1D NMR data and the typical product ion *m/z* 144.0452 (C_9_H_6_NO^+^) observed in MS/MS spectrum of **1** (Fig. S16) were indicative of a carbonyl substituent on an indole ring and revealed its close similarity to coscinamide B, a bisindole alkaloid reported from the marine sponge *Coscinoderma* sp. (25). Indeed, the only difference between **1** and coscinamide B was the saturation of the double bond at Δ^8(9)^. Thus, the olefinic protons were replaced by methylene protons (H-8 *δ*_H_ 2.95, t, *J* = 7.5 Hz and H-9 *δ*_H_ 3.52, q, *J* = 6.9 Hz) in compound **1**, to comply with the number of predicted DoUs (14; coscinamide B has 15 DoUs). This assumption was supported by the ^1^H-^1^H COSY correlations between H-8 and H-9. Further evidence came from key HMBC correlations, which were observed between H-8/C-2 (*δ*_C_ 122.7), H-8/C-3 (*δ*_C_ 111.6), H-8/3a (*δ*_C_ 127.2) and H-9/C-8 (*δ*_C_ 24.9), H-9/C-11 (*δ*_C_ 163.5), and H-9/C-3 (*δ*_C_ 111.6). The chemical structure of the saturated derivative of coscinamide B, i.e. 8,9-dihydrocoscinamide B (**1**) was unambiguously confirmed by in-depth analysis of a full set of 2D NMR experiments and HRMS data (Table S5 and Figs. S9–S16). The key COSY and HMBC correlations observed for **1** are shown in Fig. 6. Hence, compound **1** was unambiguously characterized as 8,9-dihydrocoscinamide B, a bis(indolyl)glyoxylamide alkaloid, bearing both a 3-ethyl-1H-indole and 1H-Indole-3-carbonyl moieties. 8,9-Dihydrocoscinamide B (**1**) was tested for in vitro antibacterial activity against *S. aureus* and the *ESKAPE* panel (Table S6). It was found to show moderate activity toward *S. aureus* and MRSA with IC_50_ values of 14.0 and 39.1 *µ*g/mL, respectively.

## Discussion

The deep sea is undoubtedly the most understudied habitat on Earth, this is due in part to the difficulty in accessing this environment. It holds a treasure trove of biodiversity and is a unique source of microorganisms. To date, half of the isolated species were new and certain taxa showed over 95% novelty. For this reason, numerous projects over the past decade have been funded to explore and harness the potential of the deep sea, including the EUROFLEETS PharmaDEEP project, which allowed for the collection of the sediments employed in the present study (26). For these reasons, the new deep-sea strain WUR7 (isolated in this study) was investigated through a comprehensive metabologenomics approach.

The results reported produced valuable insights at various levels. In fact, we found an unusual TDC from the deep-sea strain WUR7, which showed highly structurally conserved active sites with ZP_02040762, a TDC from the enteric strain *R. gnavus.* Their similarity revealed Trp-selectivity structural determinants, which are peculiar but of interest considering the phylogenetic distance between the two genera.

The presence of such a specific enzyme might have evolutionary rationale; indeed, microorganisms living in energy-limiting conditions such as deep-sea microorganisms over time have developed different strategies to alleviate substrate limitation—including evolving alternative catabolic systems, allowing for a wider substrate choice (27). Notably, two putative amino acid decarboxylases have been recorded in WUR7's genome.

Trp decarboxylation, which is operated by specialized TDCs, is responsible for the formation of tryptamine, which serves also as a building block in the biosynthesis of numerous plant NPs, e.g. *Catharanthus roseus*, where tryptamine is condensed with secologanin to produce a β-carboline core, employed in the biosynthesis of hundreds of alkaloids (28). Contrarily, TDCs are extremely uncommon in bacteria; they have been recently found in a few strains belonging to a small portion of the human gut microbiota, in which tryptamine is thought to play a role in the nervous system–gut microbiota relationship (13). Nevertheless, certain TDCs have been recently related to the biosynthesis of some marine microbial alkaloids, such as bacillamides C-D, physostigmine, discolins, and marinacarbolines (14–16).

Therefore, the presence of TDCs in the genome of WUR7 suggested that this enzyme might provide a tryptamine pool to be channeled in metabolic pathways, such as alkaloid biosynthesis. Therefore, despite a genome poor in SM BGCs, WUR7 might become a source of NPs by providing a specific stimulus.

To test this hypothesis, we decided to supplement WUR7 with *L*-Trp, followed by metabolomic dereplication and chemoinformatic class assignment. This workflow demonstrated that Trp addition elicited the secretion of numerous putative alkaloids. In total, 179 nodes were detected, including alkaloids and indole derivatives with numerous putative new entities, which showed no annotations on two of the major NP databases. The purification and structure elucidation of compound **1** confirmed once again our hypothesis, leading to the identification of 8,9-dihydrocoscinamide B (**1**), a bisindole alkaloid, being reported for the first time from a natural source. The unsaturated Δ^8^-analogue of compound **1**, i.e. coscinamide B, was previously isolated from the sponge *Coscinoderma* sp. (25). Considering that several *Lacinutrix* species have been isolated (or detected by functional metagenomics) from deep-sea sponge microbiota (29, 30), it can be speculated that this bacterium could be the actual producer of coscinamide B in the sponge *Coscinoderma* sp., as well as of indole-based alkaloids in other marine sponges, but this assumption has to be confirmed.

## Conclusion

Herein, we present the exploitation of the secondary metabolism of the new deep-sea strain *L. shetlandiensis* sp. nov. WUR7, through a combined approach involving genome mining, protein modeling, tailored culture media variation, and untargeted metabolomic and chemoinformatic dereplication. This strategy was successful in unlocking WUR7's hidden metabolome. Based on genomic insights, we discovered that this strain undergoes profound metabolic rewiring in response to *L*-Trp supplementation, triggering the production of numerous indole alkaloids, which would otherwise remain unproduced. This strategy enabled the isolation and chemical characterization of the antibacterial alkaloid 8,9-dihydrocoscinamide B for the first time from a natural source. In contrast with the poor SM repertoire described for this genus so far, this study demonstrated that *L. shetlandiensis* sp. nov. WUR7 can turn into an indole alkaloid factory when provided with tryptophan as stimulus. This manuscript highlights the effectiveness and the complementarity of OMIC technologies and the OSMAC approach in NP discovery, and the potential of deep-sea exploitation. Further studies will surely be dedicated to the full characterization of the remaining unknown alkaloids from WUR7.

## Materials and methods

### Sampling from the deep sea

**Fig. 7.**
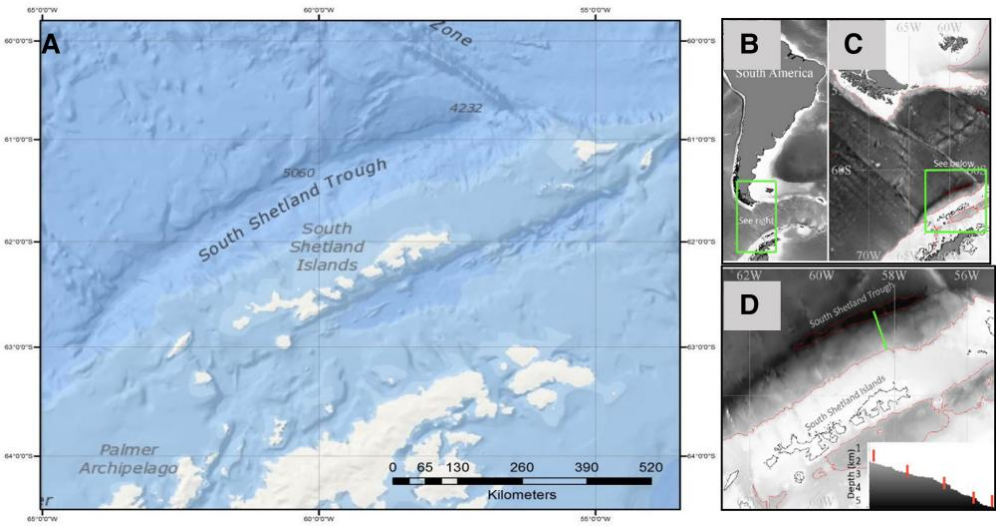
A) The South Shetland Trough, Southern Ocean, northwest of the South Shetland Islands. The location chosen for this cruise was the South Shetland Trough (SST), which runs parallel to the South Shetland Islands in Antarctica, an area 253 nautical miles long and ∼25 nm at its widest at the 4,000 m contour, and 5,200 m deep at 61°S, 59°W, which lies ∼55 nm north of King George Island. Near seafloor temperatures will decrease to near 0°C at the deepest area, where pressures exceed 52 MPa. Location of the sampling transect. B) depicts the area with reference to South America; C) shows a more detailed locality of the South Shetland Islands; D) the sampling transect line (green) from the SST's deepest point up the fore-arc toward King George Islands and (inset) the bathymetric profile of the transect line with each sampling location marked in red (personal communication, Jamieson et al., 2018).

Marine sediments were collected from a previously unexplored region (GPS: −58.035554 −61.304726) in the SST, Antarctica, using equipment on board the EU-funded EUROFLEETS2-2013, BIO Hesperides research vessel in conjunction with the PharmaDEEP (https://www.eurofleets.eu/access/previous-calls/eurofleets2-regional-2-call-results/eurofleets2-funded-project-pharmadeep-results/) project (2015) (Fig. 7)

### Bacterial isolation from deep-sea sediments

From the PharmaDEEP sediments, bacteria were isolated using culture-dependent techniques for the enrichment of marine microorganisms and cryopreserved in glycerol at −80°C. Particularly, the solid media used included MB, Nutrient Broth (NB), artificial seawater (ASW), and Sulphate-Reducing Broth (SRB) containing 10% (*w*/*v*) gellan gum as a solidifying agent. Serial dilutions were made of the respective sediments and spread plated onto the media. After ≥8 weeks of incubation at 10°C, in both aerobic and anaerobic conditions, 25 visible colonies were selected according to differences in morphology. Thereafter, the colonies were inoculated in their respective isolation (liquid) media and cultivated at 10°C for 5–10 days in agitation, followed by preparation of cryopreserved glycerol stocks, which were stored at −80°C.

More specifically, WUR7 was isolated under low temperature (10°C), long incubation periods (>8 weeks), and alternate isolation medium (MB + gellan gum).

### Strain identification, genome sequencing, assembly, and annotation

Isolate WUR7 was subjected to de novo WGS using Pacific Biosciences RS II single-molecule real-time (SMRT) technology. SMRT sequencing was followed by Illumina NextSeq 500 sequencing for error correction of the de novo–assembled genomes. Therefore, DNA was isolated using Qiagen Genomic-tip 100/G (Qiagen, Hilden Germany) according to the instructions of the manufacturer. SMRTbell template library was prepared according to the instructions from Pacific Biosciences, Menlo Park, CA, USA, following the Procedure & Checklist—Greater Than 10 kb Template Preparation. Briefly, for preparation of 15-kb libraries 8-*µ*g genomic DNA from strain WUR7 was applied unsheared. DNA was end repaired and ligated overnight to hairpin adapters applying components from the DNA/Polymerase Binding Kit P6 from Pacific Biosciences, Menlo Park, CA, USA. Reactions were carried out according to the manufacturer’s instructions. BluePippin size selection to greater than 4 kb was performed according to the manufacturer’s instructions (Sage Science, Beverly, MA, USA). Conditions for annealing of sequencing primers and binding of polymerase to purified SMRTbell template were assessed with the Calculator in RS Remote, PacificBiosciences, Menlo Park, CA, USA. 1 SMRT cell was sequenced per strain on the PacBio RSII (PacificBiosciences, Menlo Park, CA, USA) taking 240-min movies. Libraries for sequencing on Illumina platform were prepared applying the Nextera XT DNA Library Preparation Kit (Illumina, San Diego, USA) with modifications according to Baym et al. (31). Samples were sequenced on NextSeq 500. Genome assembly was performed applying the RS_HGAP_Assembly.3 protocol included in SMRT Portal version 2.3.0 applying a target genome size of 10 Mbp. The genome assembly revealed seven contigs summed up to a total genome size of 3.6 Mbp. Error correction was performed by mapping of the Illumina short reads onto finished genomes using the Burrows–Wheeler Aligner bwa 0.6.2 in paired-end (sample) mode using default setting with subsequent variant and consensus calling using VarScan 2.3.6 (32). Automated genome annotation was carried out using Prokka (33). The genome has been deposited at NCBI GenBank under accession number CP045067.

### TYGS for whole genome phylogeny

A complete genome-based taxonomic analysis was performed using the TYGS, a free bioinformatics platform available under the URL https://tygs.dsmz.de/ (34). In brief, (i) an algorithm is used to extract the highest scoring 16S rRNA gene sequence from the query genome; (ii) a BLASTn sequence comparison is then conducted against all available type strains in the database; (iii) Genome BLAST distance phylogeny (GBDP) distances are calculated between all 16S sequence pairs with a high enough bitscore; (iv) distances are sorted and *n* number of type strain genomes, most closely related to the query genome, are found; and (v) for a previously determined subset of strains, all pairwise intergenomic sequences are calculated using the GBDP. The results include a (i) genome-based phylogenetic tree, (ii) 16S rRNA gene tree, (iii) digital DNA–DNA hybridization (DDH), (iv) affiliations to (sub-) species clusters, and (v) differences in G + C content.

### Phylogenetic inference

The resulting intergenomic distances were used to infer a balanced minimum evolution tree with branch support via FASTME 2.1.4 including SPR postprocessing (30). Branch support was inferred from 100 pseudobootstrap replicates each. The trees were rooted at the midpoint and visualized with PhyD3 (35).

### Type-based species and subspecies clustering

The type-based species clustering using a 70% dDDH radius around each of the 10 type strains was done as previously applied. The resulting groups are shown in Table 2 (extended data in Table S2). Subspecies clustering was done using a 79% dDDH threshold as previously introduced (34–37).

### Genome mining using antiSMASH

The “antibiotics and SM analysis shell—antiSMASH” (https://antismash.secondarymetabolites.org)—web server was used as a tool for identifying and analyzing BGCs from WUR7 (4). Prokka annotated genome sequence data were submitted to antiSMASH 5.0 by using default parameters and incorporation of the ClusterFinder algorithm.

### Comparative enzyme modeling

The 3D structure of WUR7_ADC1 was predicted in silico by comparative analysis with the known 3D structure of a homologous TDC ZP_02040762. This was achieved by doing a homology search of WUR7_ADC1 on the PDB database. It was then modeled against the highest scoring match, “4OBV_A.pdb,” using UCSF Chimera and Modeler software packages. The quality of each of three models was assessed through PDBsum server (38). The whole ProCHECK structural analyses (34) were performed on all the models (data shown only for the best model), i.e. evaluation of conformations of residues compared to the allowed areas in the Ramachandran plot (Fig. S4). The model with higher stereo chemical quality indexes (G factor average = −0.02) was employed to create the images displayed by using the molecular graphics software VMD (39).

### Cultivation and extraction of SMs

Small-scale cultures of WUR7 were prepared by incubating it for 5 days at 20°C in 100 mL of the following media: (i) MB, (ii) MB + tryptone (15 g/L), and (iii) MB + tryptone (15 g/L) + *L*-tryptophan (20 mM). Condition 3 was upscaled to 2.1 L to enable downstream work. Bacterial cells were separated from the supernatant by centrifugation, and the supernatant was subjected to repeated solvent extraction by using ethyl acetate (EtOAc). The organic phase was then vacuum dried to generate the crude extracellular extract. The upscaled culture in condition 3 (2.1 L) was extracted as reported above and subjected to C18 SPE partitioning generating five fractions.

### LC–MS/MS analysis

For all the MS analyses, the dried samples were dissolved in MS-grade MeOH and the mobile phase was composed by a different ratio of phase A (H_2_O + 0.1% formic acid) and phase B (ACN + 0.1% formic acid). The routine LC–MS/MS profiling of the small culture extracts of WUR7 were conducted on a QTRAP 4500-Nexera X2 UHPLC equipped with a C18 column and operated at a flow rate of 0.2 mL/min with the following gradient: initial 90% A—10% B; 0–15 min, 0% A—100% B; and 15–20 min 0% A—100% B. LC–HRMS/MS analyses were carried on a Xevo G2-XS QToF-UPLC I-Class System. The gradient was set as follows: initial, 99% A—1% B; 0–11.5 min, 1% A—99% B; and 11.5–14.5 min 0% A—100% B, equipped with a C18 column. MeOH and the extracted culture media were employed as negative controls, and all the features they contained were deleted in all the samples. Each sample was run in duplicate.

### Molecular networking and chemoinformatic analysis

The .raw files were converted into .mzXML files by using MSConvert, they were processed with MZmine (40) and submitted to the GNPS to build a FBMN (2, 41), and MolNetEnhancer workflow (42) and Dereplicator Plus (43) were also employed and integrated in the Network. Molecular Networks were visualized on Cytoscape (44) where redundancies were manually deleted. FBMN and MolNetEnhancer jobs are publicly accessible at the respective links: https://gnps.ucsd.edu/ProteoSAFe/status.jsp?task=f52418c813d74418961f94d1d7304716 and https://gnps.ucsd.edu/ProteoSAFe/status.jsp?task=67d8a632d6f746bbbeb6b495ecaab82d.

### HPLC purification

The initial HPLC separation was carried out using a semipreparative C18 column connected to a Jasco HPLC and revealed with a photodiode array detector. The mobile phase was composed of different ratios of Buffer A (100% H_2_O + 0.1% TFA) and Buffer B (100% Acetonitrile + 0.1% TFA) at a flow rate of 2.00 mL/min. The following gradient was employed: initial 75% A—25% B; 0–37 min, 15% A—85% B; and 37–40 min 0% A—100% B. P4 was further purified on an analytical PFP column, using the gradient: initial 75% A—25% B and 0–10 min, 0% A—100% B, operating at 1 mL/min as flow, to afford 1.5 mg of pure compound **1**.

### NMR characterization

Compound **1** was dissolved in 350 *µ*L of DMSO-*d_6_* and transferred into a 5.0 mm Shigemi tube. NMR spectra were recorded on a Bruker AV 600 spectrometer (600 and 150 MHz for ^1^H and ^13^C NMR, respectively). The residual solvent signals for DMSO-*d_6_* (*δ*_H_ 2.50 and *δ*_C_ 39.51 ppm) were used as internal references. Data analyses were performed with MestReNova.

### Compound 1 description

Compound **1**: 8,9-Dihydrocoscinamide B (*N-(2-(1H-Indol-3-yl)ethyl)-2-(1H-indol-3-yl)-2-oxoacetamide*) (**1**): white amorphous powder; ^1^H (600 MHz) and ^13^C (150 MHz) NMR data, (Table S5); HR-ESIMS *m/z* 354.1219 [M + Na]+ (calcd for C_20_H_17_N_3_O_2_Na, 354.1219) (Fig. S15).

### Antimicrobial assay

The antimicrobial activity of 8,9-dihydrocoscinamide B was assessed by liquid inhibition assay in 96-well plates against *S. aureus* and the *ESKAPE* panel (*Enterococcus faecium*, MRSA, *Klebsiella pneumoniae*, *Acinetobacter baumannii*, *Pseudomonas aeruginosa*, and *Escherichia coli*) as previously described (45).

## Supplementary Material

pgad221_Supplementary_Data

## Data Availability

Authors declare that the data supporting the findings reported in this study are openly available in public repositories. Fractions and extracts mass spectrometry mzml data are respectively available on MassIVE repository at the following links ftp://MSV000092278@massive.ucsd.edu and ftp://MSV000092280@massive.ucsd.edu; FBMN and MolNetEnhancer jobs are publicly accessible at the respective links: https://gnps.ucsd.edu/ProteoSAFe/status.jsp?task=f52418c813d74418961f94d1d7304716 and https://gnps.ucsd.edu/ProteoSAFe/status.jsp?task=67d8a632d6f746bbbeb6b495ecaab82d, while the genome has been deposited at NCBI GenBank under the accession number CP045067.
