# Supplementary material for: A metabologenomics approach to unlock the metabolome of the novel Antarctic deep-sea isolate Lacinutrix shetlandiensis sp. nov. WUR7
Source: PNAS Nexus. 2023 Jul 6;2(7):pgad221. doi: 10.1093/pnasnexus/pgad221 (PMC10337856; doi:10.1093/pnasnexus/pgad221)
Supplement: pgad221_Supplementary_Data [file pgad221_supplementary_data.docx]

**
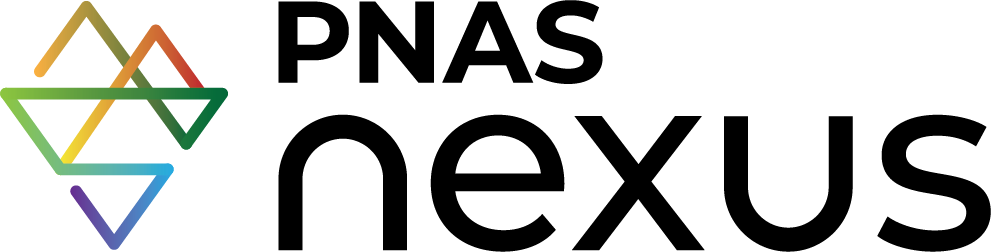
**

**Supporting Information for**

A metabologenomics approach to unlock the metabolome of the novel Antarctic deep-sea isolate *Lacinutrix shetlandiensis* sp. nov. WUR7

Giovanni A. Vitale ^a,b,e,†^, Grant G. January ^c,e,†^, Ernest Oppong-Danquah ^d^, Gerardo Della Sala ^b^*, Fortunato Palma Esposito ^b,e^, Deniz Tasdemir ^d,f^ and Donatella de Pascale ^b,e^*

^a^ CMFI Cluster of Excellence, Interfaculty Institute of Microbiology and Medicine, University of Tübingen, Tübingen 72076, Germany

^b^ Department of Eco-sustainable Marine Biotechnology, Stazione Zoologica Anton Dohrn (SZN), Giardini Molosiglio Via Acton 55, I-80121 Naples, Italy

^c^ Derriford Research Facility, School of Biomedical Sciences, Faculty of Health, University of Plymouth, 14 Research Way, PL6 8BU, Plymouth, United Kingdom

^d^ GEOMAR Centre for Marine Biotechnology (GEOMAR-Biotech), Research Unit Marine Natural Products Chemistry, GEOMAR Helmholtz Centre for Ocean Research Kiel, Am Kiel-Kanal 44, 24106 Kiel, Germany

^e^ Institute of Biochemistry and Cell Biology, National Research Council, 80131 Naples, Italy

^f^ Kiel University, Christian-Albrechts-Platz 4, 24118 Kiel, Germany, ORCID ID: [0000-0002-7841-6271](callto:0000-0002-7841-6271)

* Donatella de Pascale, Gerardo Della Sala

^†^ Equally Contributed

**Email:**  [donatella.depascale@szn.it](mailto:donatella.depascale@szn.it), gerardo.dellasala@szn.it

**This PDF file includes:**

Tables S1 to S6

Figures S1 to S16

Extended methods

SI References

**Table S1.** Complete genome statistics of *Lacinutrix shetlandiensis* sp. nov. WUR7.

|  |  | **Number** | **% Of Total** |
| **DNA, total number of bases** |  | 3987475 | 100.00% |
| DNA coding number of bases |  | 3600562 | 90.30% |
| DNA G+C number of bases |  | 1296092 | 32.50% |
|  |  |  |  |
| **DNA scaffolds** |  | 1 | 100.00% |
| CRISPR Count |  | 1 |  |
| **Genes total number** |  | 3559 | 100.00% |
| Protein coding genes |  | 3449 | 96.91% |
| Regulatory and miscellaneous features |  | 52 | 1.46% |
| RNA genes |  | 58 | 1.63% |
| rRNA genes |  | 12 | 0.34% |
| 5S rRNA |  | 4 | 0.11% |
| 16S rRNA |  | 4 | 0.11% |
| 23S rRNA |  | 4 | 0.11% |
| tRNA genes |  | 43 | 1.21% |
| Other RNA genes |  | 3 | 0.08% |
| Protein coding genes with function prediction |  | 2476 | 69.57% |
| without function prediction |  | 973 | 27.34% |
| Protein coding genes with enzymes |  | 772 | 21.69% |
| Protein coding genes connected to KEGG pathways |  | 750 | 21.07% |
| not connected to KEGG pathways |  | 2699 | 75.84% |
| Protein coding genes connected to KEGG Orthology (KO) |  | 1348 | 37.88% |
| not connected to KEGG Orthology (KO) |  | 2101 | 59.03% |
| Protein coding genes connected to MetaCyc pathways |  | 671 | 18.85% |
| not connected to MetaCyc pathways |  | 2778 | 78.06% |
| Protein coding genes with COGs |  | 2419 | 67.97% |
| with Pfam |  | 2610 | 73.34% |
| with TIGRfam |  | 968 | 27.20% |
| with SMART |  | 670 | 18.83% |
| with SUPERFam |  | 2968 | 83.39% |
| with CATH FunFam |  | 2338 | 65.69% |
| in internal clusters |  | 543 | 15.26% |
| in Chromosomal Cassette |  | 3528 | 99.13% |
| Chromosomal Cassettes |  | 214 | - |
| Protein coding genes coding signal peptides |  | 495 | 13.91% |
| Protein coding genes coding transmembrane proteins |  | 835 | 23.46% |
|  |  |  |  |
|  |  |  |  |
| **COG clusters** |  | 1457 | 60.23% |
| **KOG clusters** |  |  | 0.00% |
| **Pfam clusters** |  | 1806 | 69.20% |
| **TIGRfam clusters** |  | 745 | 76.96% |

**Table S2.** Pairwise comparisons of *Lacinutrix shetlandiensis* WUR7 genome vs. 10 of the closest related type**-**strain genomes.

| **Subject strain** | **dDDH (d0,%)** | **C.I. (d0,%)** | **dDDH (d4,%)** | **C.I. (d4,%)** | **dDDH (d6,%)** | **C.I. (d6,%)** | **G+C content difference (%)** |
| --- | --- | --- | --- | --- | --- | --- | --- |
| ***Lacinutrix himadriensis* E4-9a** | 69.5 | [65.6 - 73.1] | **50.4** | **[47.8 - 53.0]** | 67.1 | [63.7 - 70.3] | 0.1 |
| *Lacinutrix algicola* AKS293 | 18.4 | [15.3 - 22.0] | 22.6 | [20.3 - 25.1] | 18.2 | [15.5 - 21.2] | 1.14 |
| *Lacinutrix mariniflava* AKS432 | 17.6 | [14.5 - 21.1] | 22.2 | [20.0 - 24.7] | 17.4 | [14.8 - 20.4] | 0.73 |
| *Oceanihabitans sediminis* DSM 28133 | 21.4 | [18.2 - 25.0] | 21.9 | [19.7 - 24.4] | 20.5 | [17.8 - 23.6] | 0.61 |
| *Olleya namhaensis* DSM 28881 | 16.6 | [13.6 - 20.1] | 21.4 | [19.2 - 23.9] | 16.5 | [13.9 - 19.4] | 0.16 |
| *Winogradskyella epiphytica* KCTC 12220 | 13.7 | [10.9 - 17.0] | 21 | [18.8 - 23.4] | 14 | [11.6 - 16.8] | 1.67 |
| *Lacinutrix jangbogonensis* PAMC 27137 | 16 | [13.0 - 19.4] | 20.8 | [18.5 - 23.2] | 16 | [13.4 - 18.9] | 0.32 |
| *Olleya marilimosa* CAM030 | 16.8 | [13.8 - 20.3] | 20.5 | [18.3 - 23.0] | 16.7 | [14.1 - 19.6] | 0.7 |
| *Winogradskyella undariae* CCUG 63832 | 14.7 | [11.8 - 18.1] | 20.5 | [18.3 - 22.9] | 14.9 | [12.4 - 17.7] | 0.73 |
| *Psychroserpens burtonensis* DSM 12212 | 14.1 | [11.3 - 17.5] | 20.5 | [18.3 - 22.9] | 14.4 | [11.9 - 17.2] | 0.91 |
| *Olleya sediminilitoris* YSTF-M6T | 16.7 | [13.7 - 20.2] | 20.5 | [18.3 - 23.0] | 16.6 | [14.0 - 19.5] | 1.43 |
| *Algibacter lectus* DSM 15365 | 14.4 | [11.6 - 17.8] | 20.3 | [18.1 - 22.8] | 14.6 | [12.1 - 17.4] | 0.81 |
| *Lacinutrix venerupis* DSM 28755 | 17 | [14.0 - 20.5] | 20 | [17.8 - 22.5] | 16.8 | [14.2 - 19.7] | 2.06 |
| *Olleya aquimaris* DSM 24464 | 16.3 | [13.3 - 19.8] | 19.6 | [17.4 - 22.0] | 16.2 | [13.6 - 19.1] | 0.26 |
| *Seonamhaeicola algicola* Gy8 | 14 | [11.2 - 17.3] | 19.5 | [17.3 - 21.9] | 14.2 | [11.8 - 17.0] | 0.22 |

**These disclosed Lacinutrix himadriensis E4-9a(T) as the closest related type strain to L. shetlandiensis sp. nov. WUR7. The probability that this value is correct was confirmed by the confidence interval that was between 47.8 – 53.0% by linear regression.*

**Table S3.** Biosynthetic gene cluster (BGCs) annotation of *Lacinutrix shetlandiensis* sp. nov. WUR7. The whole genome of WUR7 has been mined for secondary metabolite gene clusters by using the antiSMASH bioinformatic tool (<https://antismash.secondarymetabolites.org>), shedding light on the presence of only 3 BGCs.

| Region | Type | From | To | Most similar BGC | Similarity score - procluster to region | Similarity score - region to region | Organism |
| --- | --- | --- | --- | --- | --- | --- | --- |
| 1 | Arylpolyen, resorcinol | 1431360 | 1485114 | flexirubin | 0.78 | 0.73 | *Flavobacterium johnsoniae UW101* |
|  |  |  |  |  |  |  |  |
| 2 | Terpene | 1764970 | 1785806 | flexixanthin | 0.25 | 0.58 | *Algoriphagus sp. KK10202C* |
|  |  |  |  |  |  |  |  |
| 3 | ladderane | 3387254 | 3428402 | naphtocyclinone | 0.16 | 0.47 | *Streptomyces arenae* |

**Table S4.** Annotated metabolites from extracellular molecular network of *Lacinutrix shetlandiensis* sp. nov. WUR7. The annotation was carried out by a combination of GNPS and DNP outputs.

| **Chemical classification** | **R_t_** (min) | **Precursor mass** | **Error**  (ppm) | | **Cluster index** | **DNP ID** | **GNPS ID** | **Analogue modification** |
| --- | --- | --- | --- | --- | --- | --- | --- | --- |
|  | **Alkaloids and indole derivatives** | | | | | | | |
| Indoles and derivatives | 4.51 | 203.1183 | -0.68 | 7704 | |  | *N*-acetyl tryptamine (Gold) |  |
| Indoles and derivatives | 6.15 | 245.1653 | -0.36 | 7715 | | *N*-pentanoyl tryptamine |  |  |
| Indoles and derivatives | 1.84 | 177.1024 | -2.19 | 292 | |  | Serotonin (Gold) |  |
| Indoles and derivatives | 3.00 | 169.0765 | -0.43 | 8997 | | Norharmane | Norharmane |  |
| Indoles and derivatives | 2.21 | 203.1182 | -1.17 | 1952 | | Shepherdine |  |  |
| Alkaloids | 7.07 | 332.1396 | -0.91 | 8607 | | 8,9-dihydrocoscinamide B |  |  |
| Alkaloids | 4.59 | 288.1495 | -1.99 | 369 | | Naufoline |  |  |
|  | **Analogues** | | | | | | | |
| Indoles and derivatives | 4.40 | 189.1025 | -1.52 | 2208 | |  | *N*-formyl tryptamine | (-CH2) |
| Indoles and derivatives | 5.03 | 217.1338 | -1.33 | 7715 | |  | *N*-propanoyl tryptamine | (+CH2) |
| Indoles and derivatives | 3.20 | 245.1651 | -1.18 | 8134 | | Shepherdine analogue |  | (+C3H8) |

**Table S5.** NMR data of compound **1** and coscinamide B (**1**) (1) acquired in DMSO-*d*_6_.

| Position | Compound 1 | | Coscinamide B | |
| --- | --- | --- | --- | --- |
|  | *δ*_C_, Type (150 MHz) | *δ*_H_, Mult. (*J* in Hz)  (600 MHz) | *δ*_C_, Type  (125 MHz) | *δ*_H_, Mult. (*J* in Hz)  (500 MHz) |
| 1-NH |  | 10.82 br s |  | 11.2 br s |
| 2 | 122.7, CH | 7.20 d (2.0) | 124.3, CH | 7.49 s |
| 3 | 111.6, C |  | 111.6, C |  |
| 3a | 127.2, C |  | 124.8, C |  |
| 4 | 118.3, CH | 7.60 d (7.8) | 119.0, C | 7.71 d (7.5) |
| 5 | 118.2, CH | 6.98 dd (7.2, 7.8) | 119.4, CH | 7.10 dd (7.5,7.5) |
| 6 | 120.9, CH | 7.06 dd (7.2, 7.8) | 121.6, CH | 7.15 dd (7.5,7.5) |
| 7 | 111.4, CH | 7.34 d (8.0) | 111.9, CH | 7.40 d (7.5) |
| 7a | 136.3, C |  | 136.9, C |  |
| 8 | 24.9, CH_2_ | 2.95 t (7.5) | 109.99, CH | 6.87 d (15) |
| 9 | 39.4, CH_2_ | 3.52 q (6.9) | 118.6, CH | 7.45 dd (10,15) |
| 10-NH |  | 8.81 t (6.0) |  | 10.82 d (10) |
| 11 | 163.5, C |  | 160.3, C |  |
| 12 | 182.2, C |  | 181.1, C |  |
| 13 | 112.2, C |  | 112.3, C |  |
| 14 | 138.5, CH | 8.75 s | 138.6, C | 8.86 s |
| 15-NH |  | 12.22 s |  | 12.26 br s |
| 15a | 136.3, C |  | 136.3, C |  |
| 16 | 112.6, CH | 7.53 d (6) | 112.6, CH | 7.56 m |
| 17 | 123.4, CH | 7.26 m | 123.5, CH | 7.28^b^ m |
| 18 | 122.6, CH | 7.25 m | 122.6, CH | 7.29^b^ m |
| 19 | 121.3, CH | 8.22 d (7.2) | 121.3, CH | 8.3 m |
| 19a | 126.2, C |  | 126.2, C |  |

**Table S6.** *In vitro* antimicrobial activity results of 8,9-dihydrocoscinamide B (**1**).

|  | IC_50_ [µg/mL]^a^ | | | | | | |
| --- | --- | --- | --- | --- | --- | --- | --- |
|  | Sa | MRSA | Efm | Kp | Ab | Psa | Ec |
| **1** | 14.0 (± 0.1) | 39.1 (± 1.4) | > 200 | > 200 | > 200 | > 200 | > 200 |
| Positive control | 1.0 (± 0.1) | 1.6 (± 0.1) | 0.2 (± 0.0) | 3.0 (± 0.2) | 0.01 (± 0.0) | 0.3 (± 0.0) | 6.4 (± 0.8) |

^a^ Positive controls: Chloramphenicol for *Staphylococcus aureus* (Sa), methillicin resistant *Staphylococcus aureus* (MRSA), *Klebsiella pneumoniae* (Kp), and *Escherichia coli* (Ec); Ampicillin for *Enterococcus faecium* (Efm); Doxycycline for *Acinetobacter baumannii* (Ab); Polymyxin B for *Pseudomonas aeruginosa* (Psa).


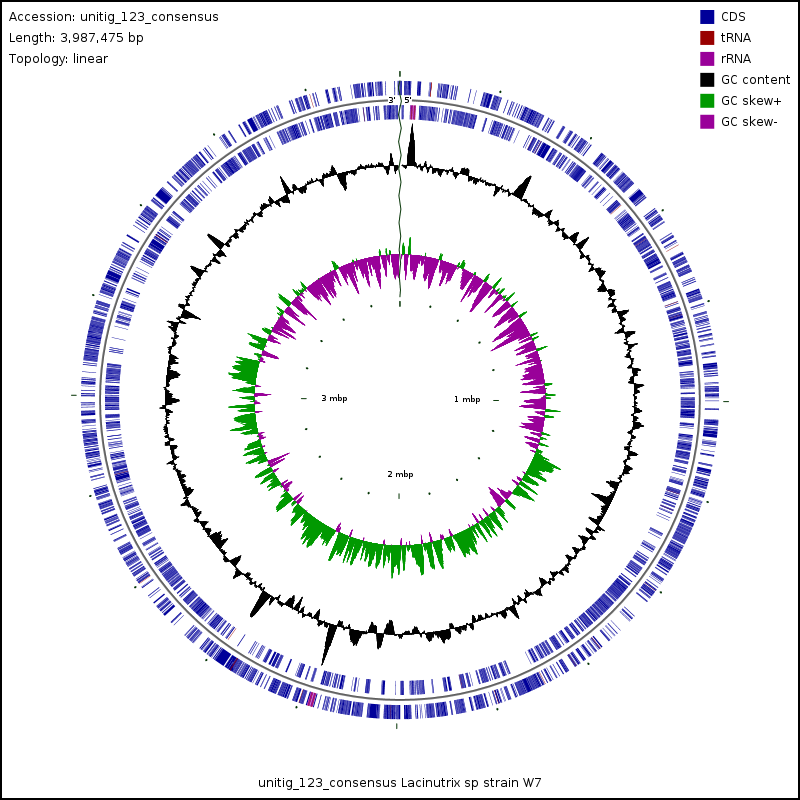


**Fig. S1.** Visualisation of *Lacinutrix shetlandiensis* sp. nov. WUR7 genome. The blue rings represent the coding sequences (CDS), tRNA in red, rRNA in purple, GC content in black, GC skew + in green, and GC skew – in purple. The *de novo* WGS of WUR7 yielded a circular consensus sequence of 1 chromosome of 3,987,475 bp, 0 plasmids, a G+C content of 32.5% and 3506 protein coding genes (Table 2). WUR7 sequenced and annotated genome is the most complete *Lacinutrix* genome to date with 125X coverage, as a hybrid sequencing approach was used, utilising 3^rd^ generation DNA sequencing technology (PacBio) and 2^nd^ generation DNA sequencing technology (Illumina) to correct for its errors.


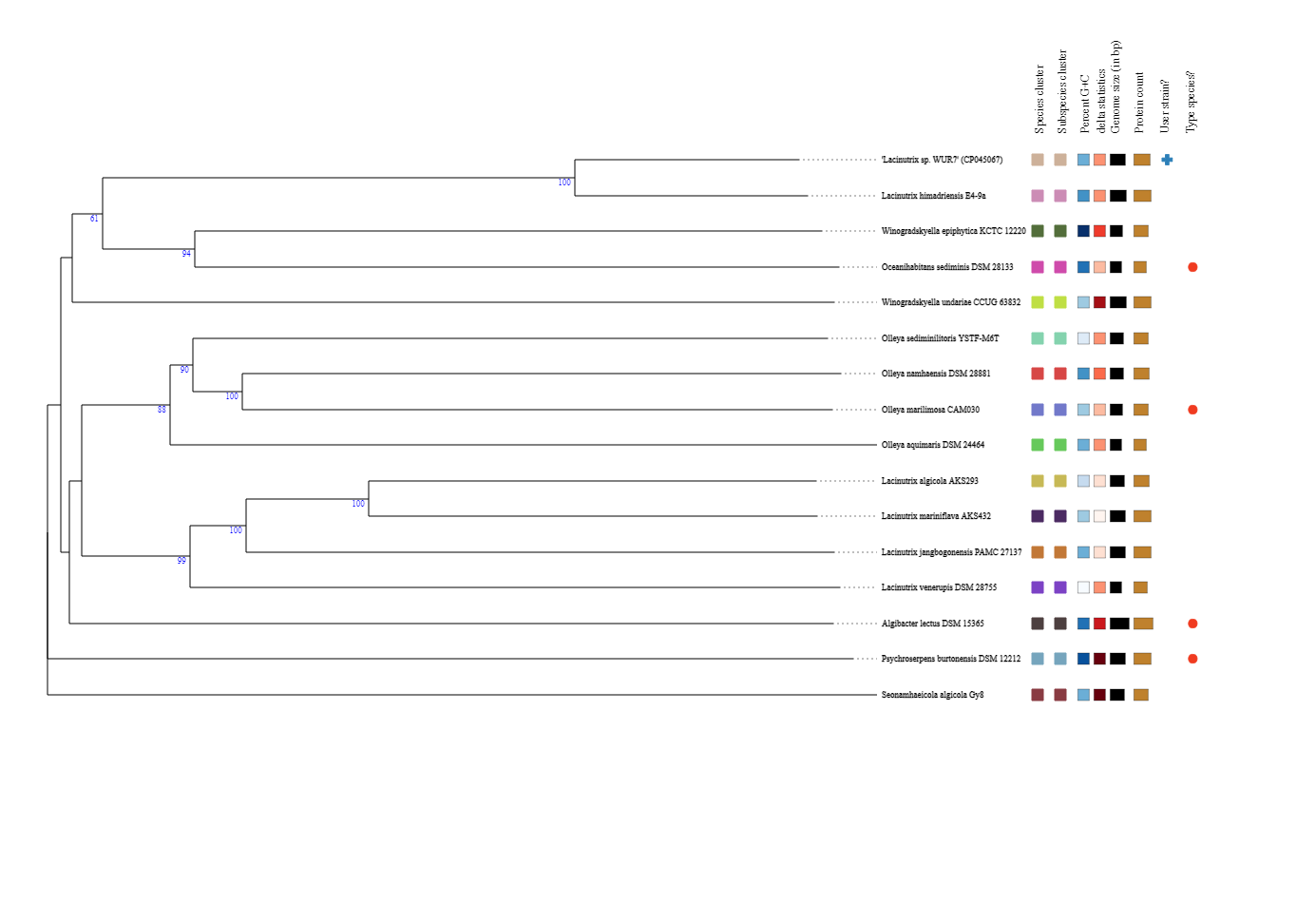


**Fig. S2.** GBDP phylogeny by WGS data of *Lacinutrix* *shetlandiensis* sp. nov. WUR7. The phylogenetic tree has been inferred from GBDP distances calculated from genome sequences within the Type Strain Genome Server ([tygs.dsacemz.de](about:blank)). The numbers above the branches are GBDP pseudo-bootstrap support values from 100 replications, with an average branch support of 63.6 %. The tree was rooted at the midpoint. The upper part of this view displays information on the Genome BLAST Distance Phylogeny (GBDP) formula and algorithm. Additional annotation is displayed to the right-hand side of the interactive tree viewer including genome-based species and subspecies clusters, genomic G+C content (in %), total sequence length (in bp), number of proteins and an indication whether the strain was provided by the user. Branch lengths are scaled in terms of the used GBDP distance formula.


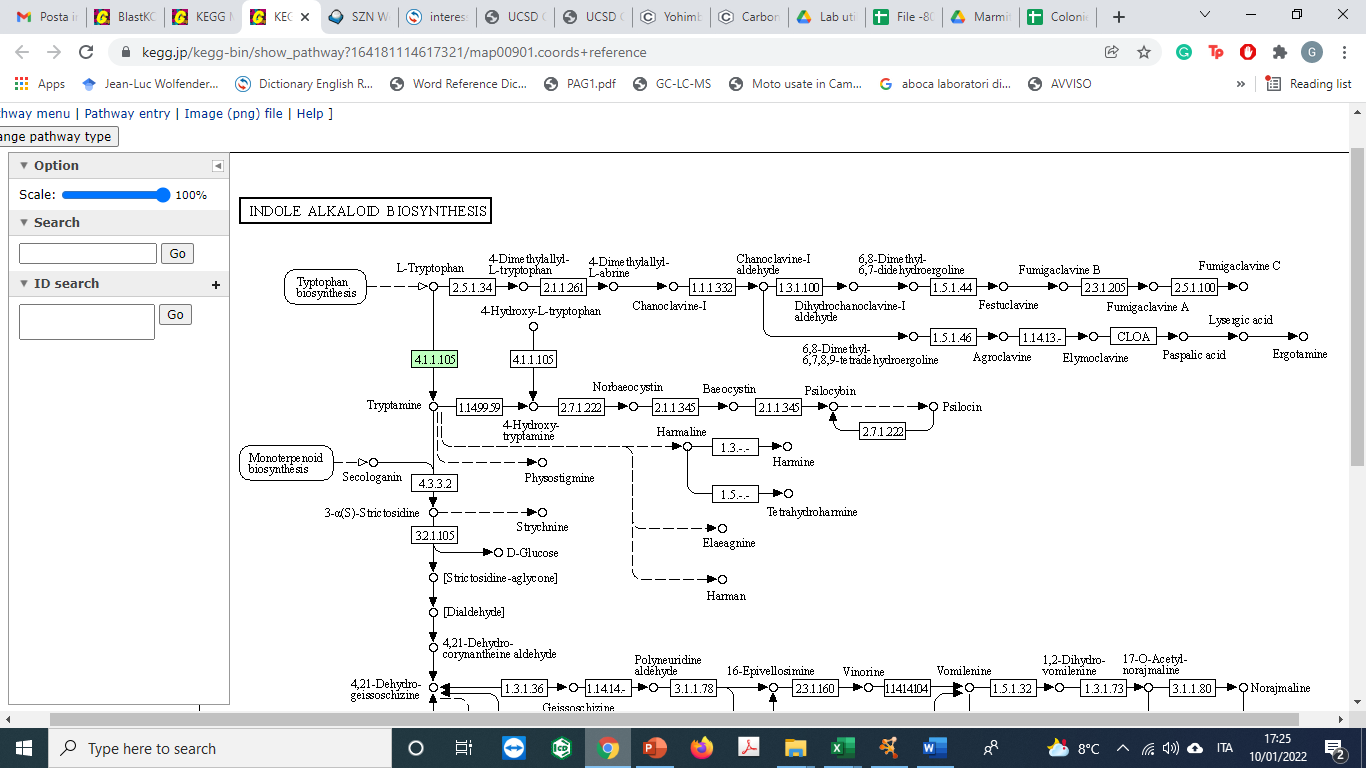


**Fig. S3.** Overview of the indole alkaloid biosynthetic pathways as annotated by BlastKoala from the genome of *Lacinutrix shetlandiensis* sp. nov. WUR7.

.


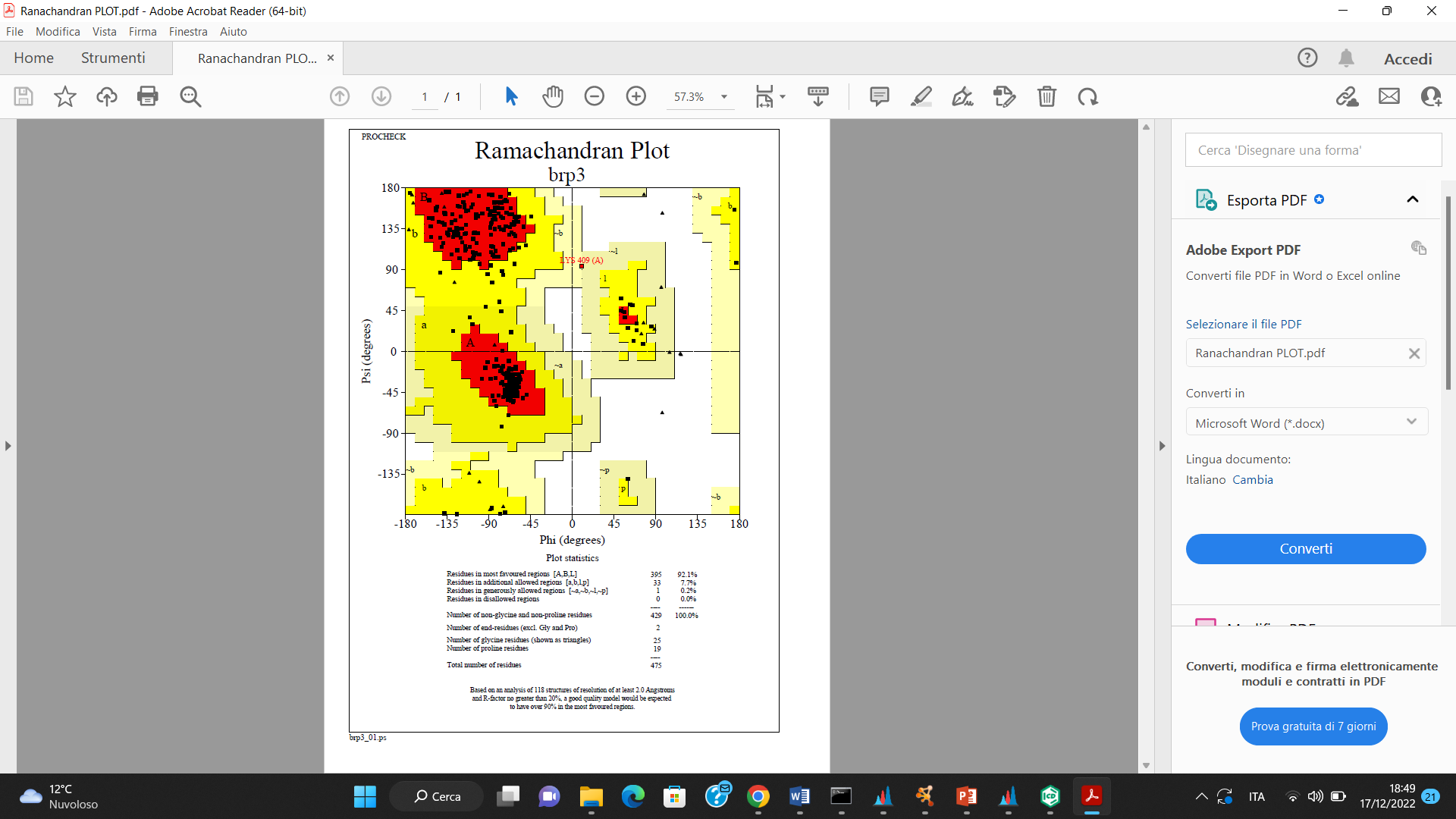


**Fig. S4**. Ramachandran Plot and statistics of WUR7_ADC1 model adopted for the superimposition.

**Fig. S5.** Putative fragmentation pathway of **A**) N-(2-(1H-indol-3-yl)ethyl)pentanamide, **B)** N-(2-(1H-indol-3-yl)ethyl)acetamide analogue at *m/z* 217.1338, and **C)** N-(2-(1H-indol-3-yl)ethyl)acetamide analogue at *m/z* 189.1025.
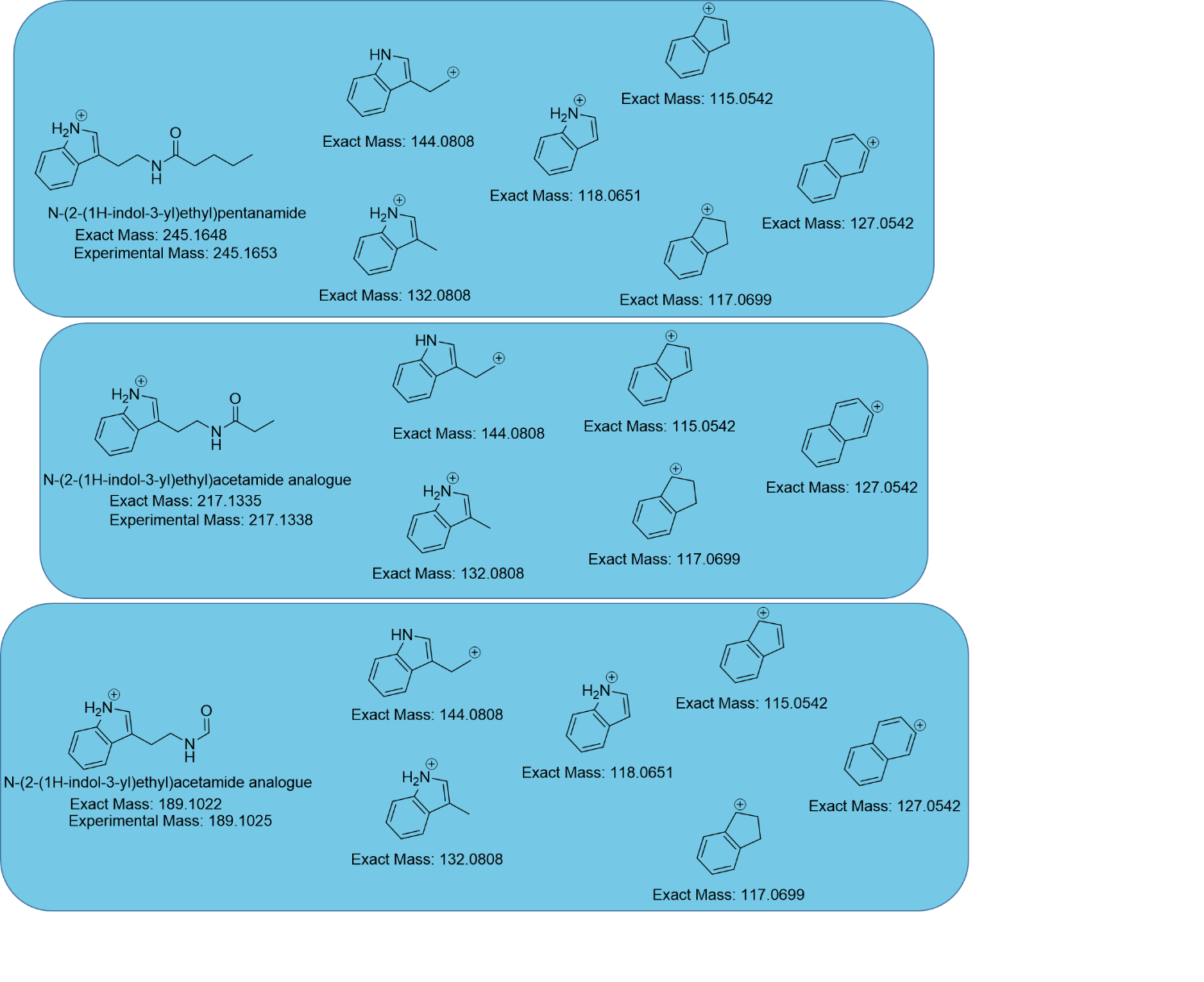


**A**

**B**

**C**


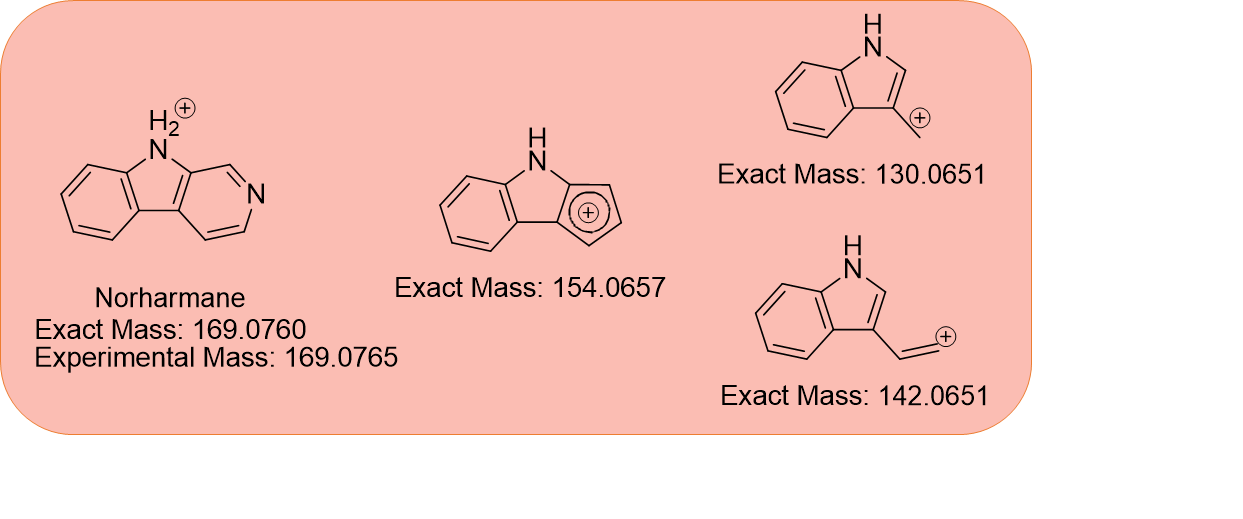


**Fig. S6.** Putative fragmentation pathway of norharmane.


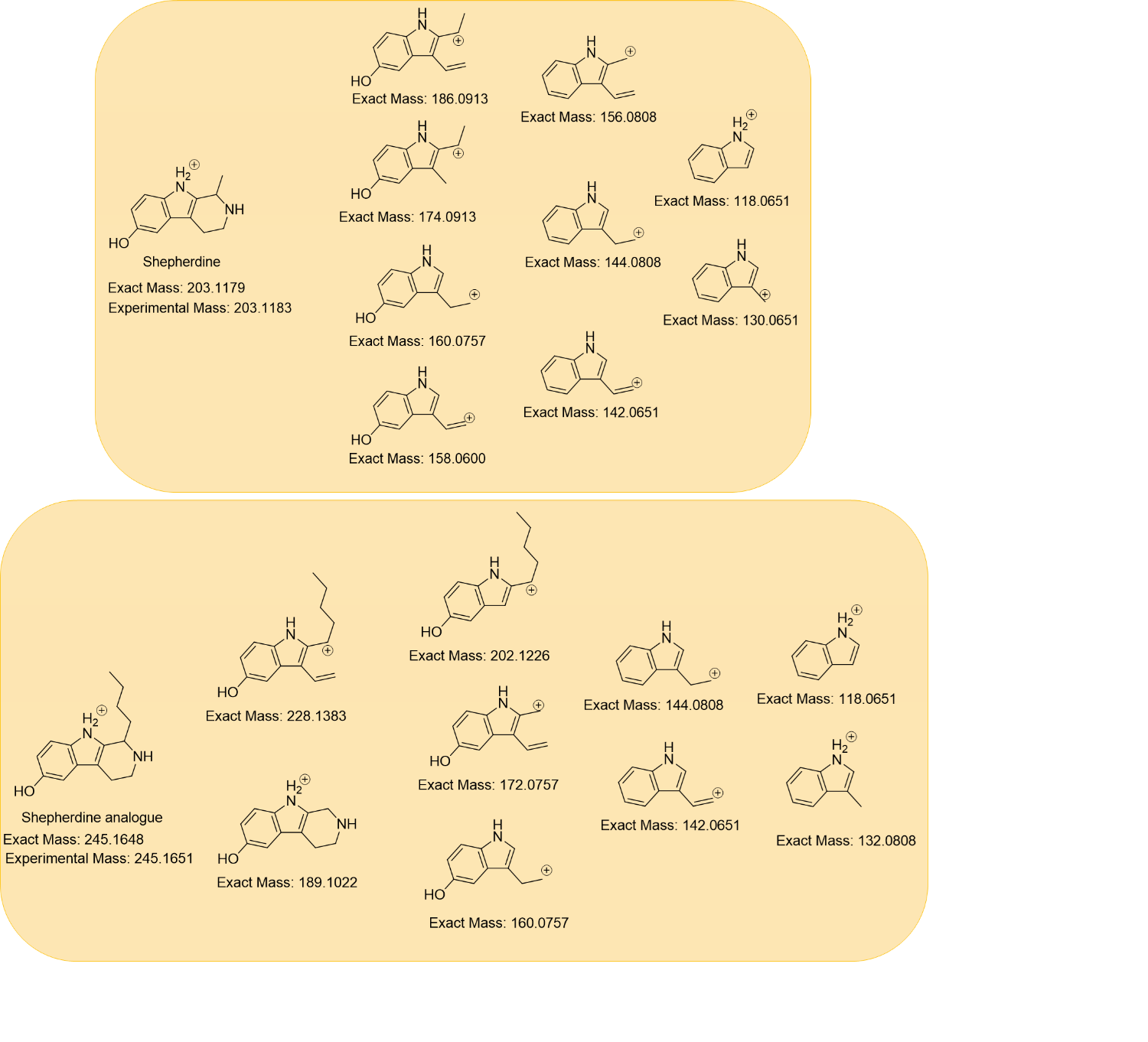


**A**

**B**

**Fig. S7.** Putative fragmentation pathway of **A)** shepherdine and **B)** its analogue with *m/z* 245.1651.


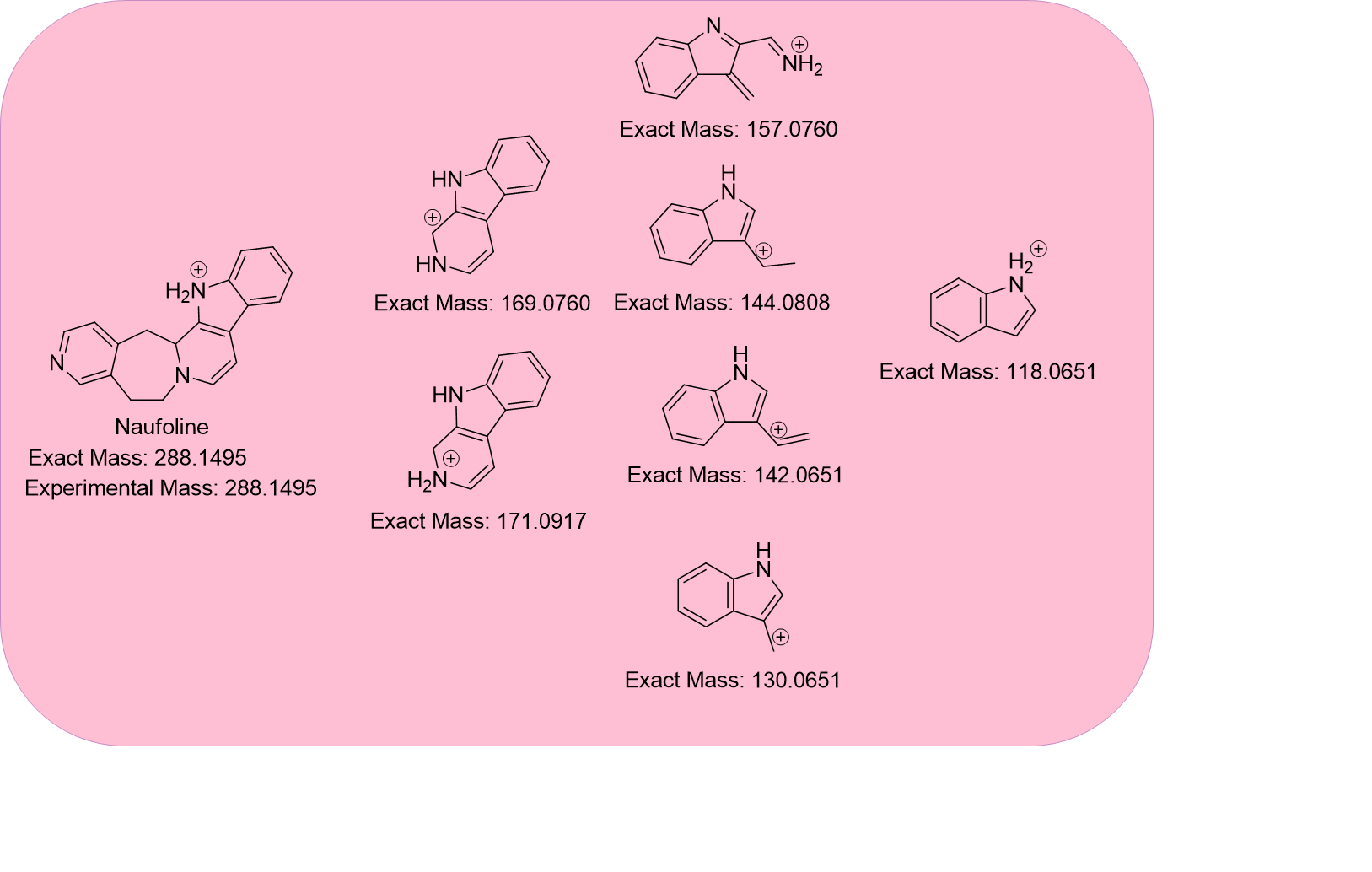


**Fig. S8**. Putative fragmentation pathway of naufoline.


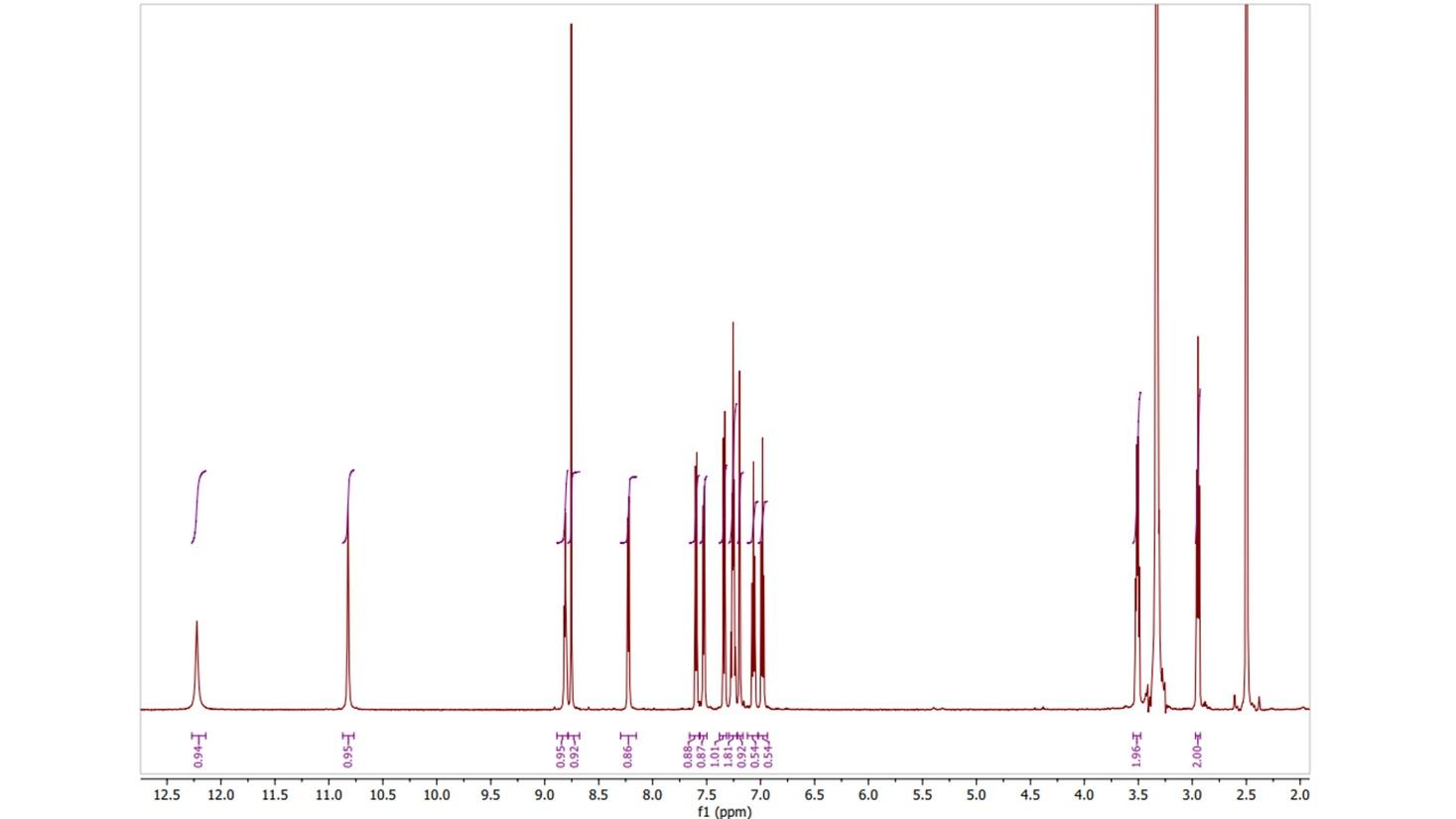


**Fig. S9.** ^1^H NMR spectrum of compound **1** (DMSO-*d*_6_, 600 MHz).


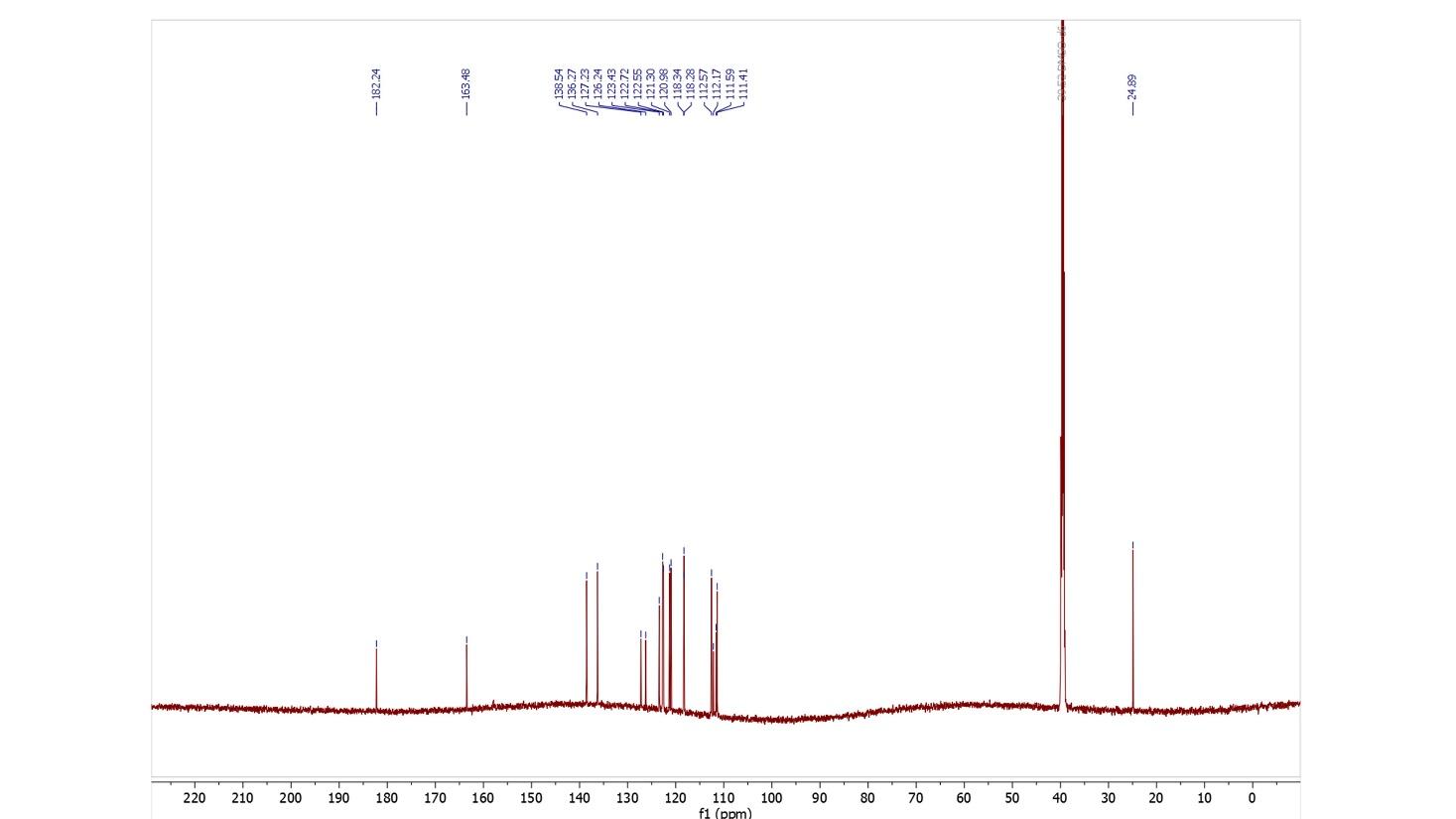


**Fig. S10.** ^13^C NMR spectrum of compound **1** (DMSO-*d*_6_, 150 MHz).


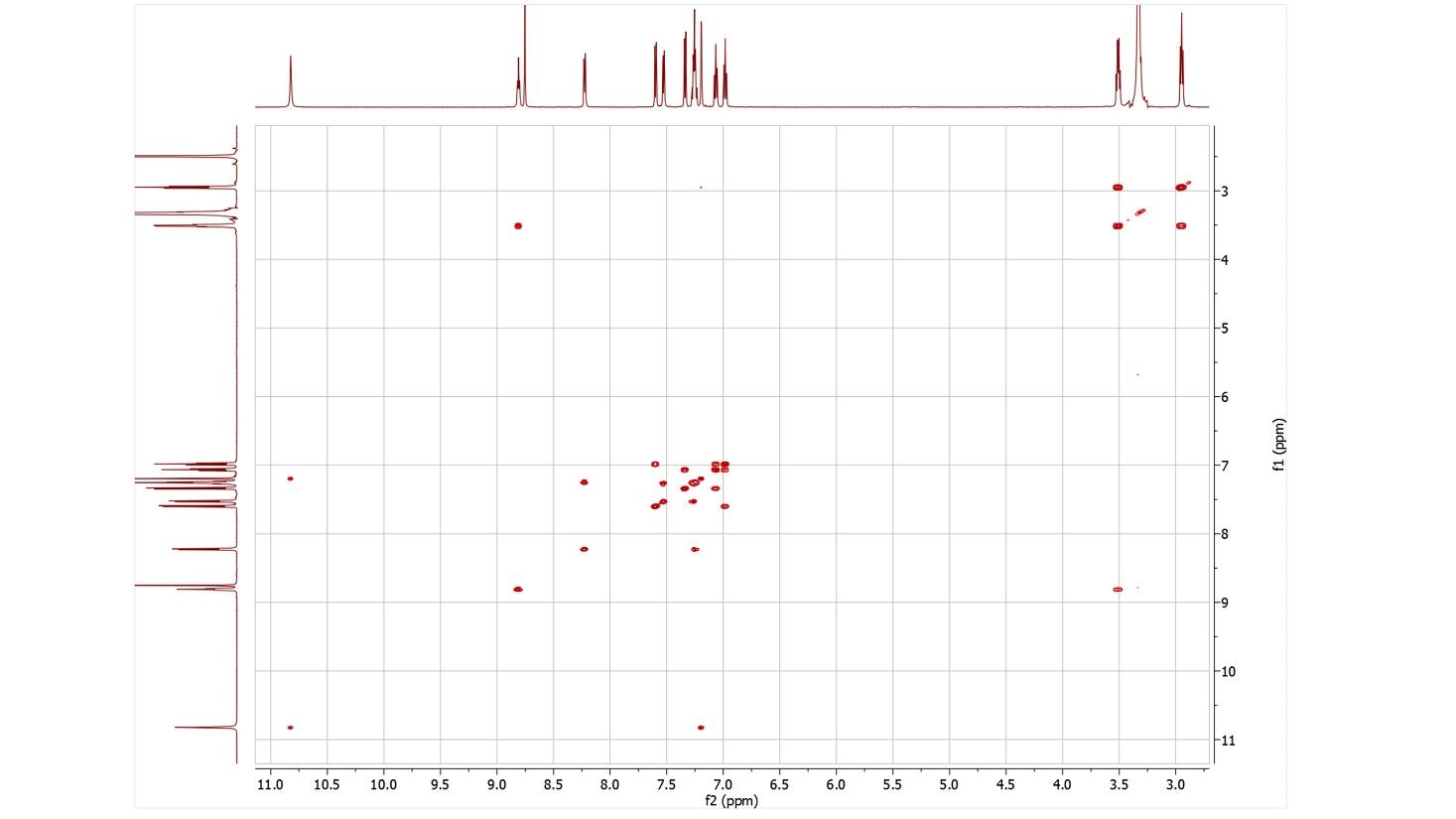


**Fig.** **S11.** COSY spectrum of compound **1** (DMSO-*d*_6_, 600 MHz).


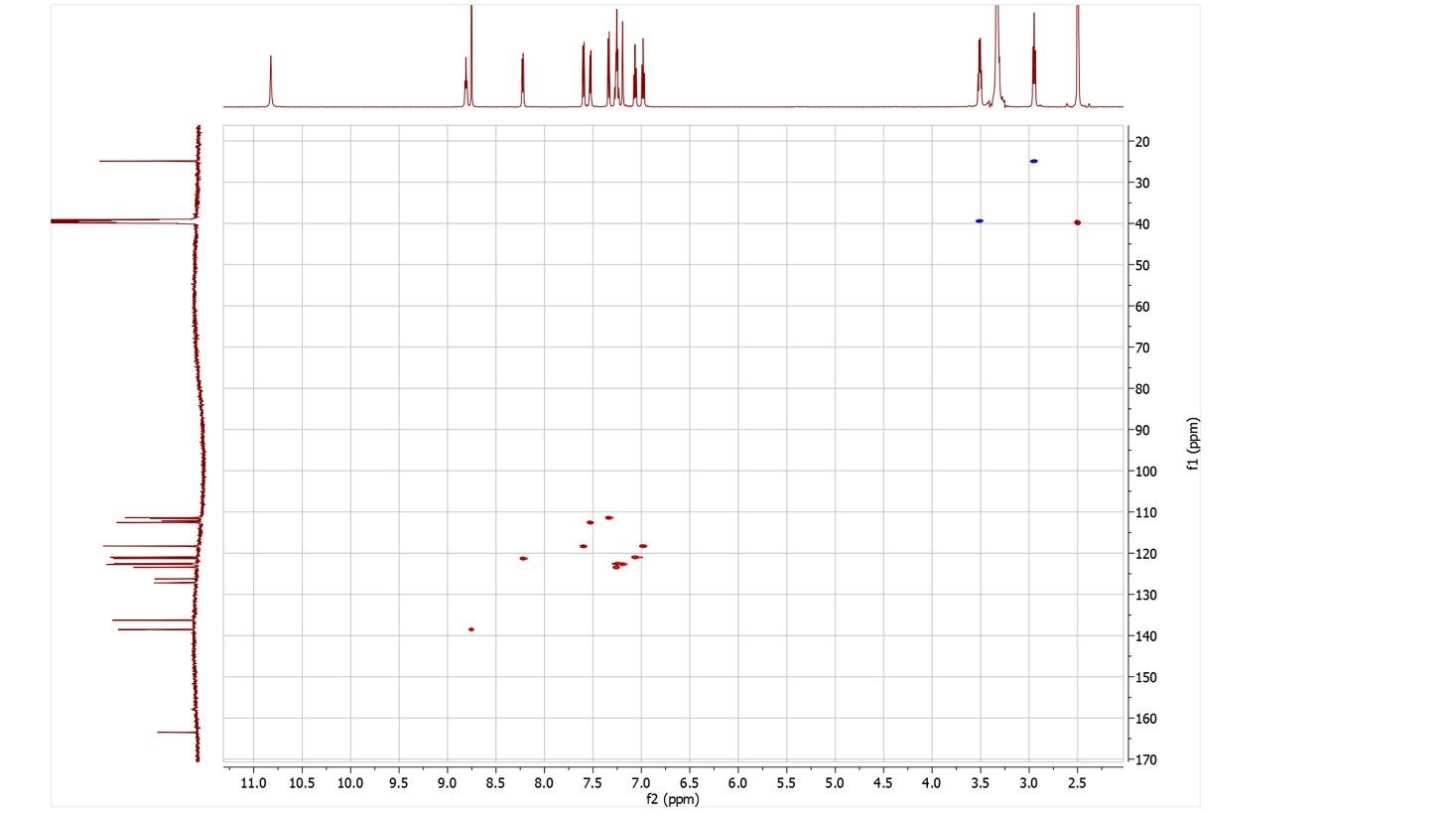


**Fig. S12.** HSQC spectrum of compound **1** (DMSO-*d*_6_, 600/150 MHz).


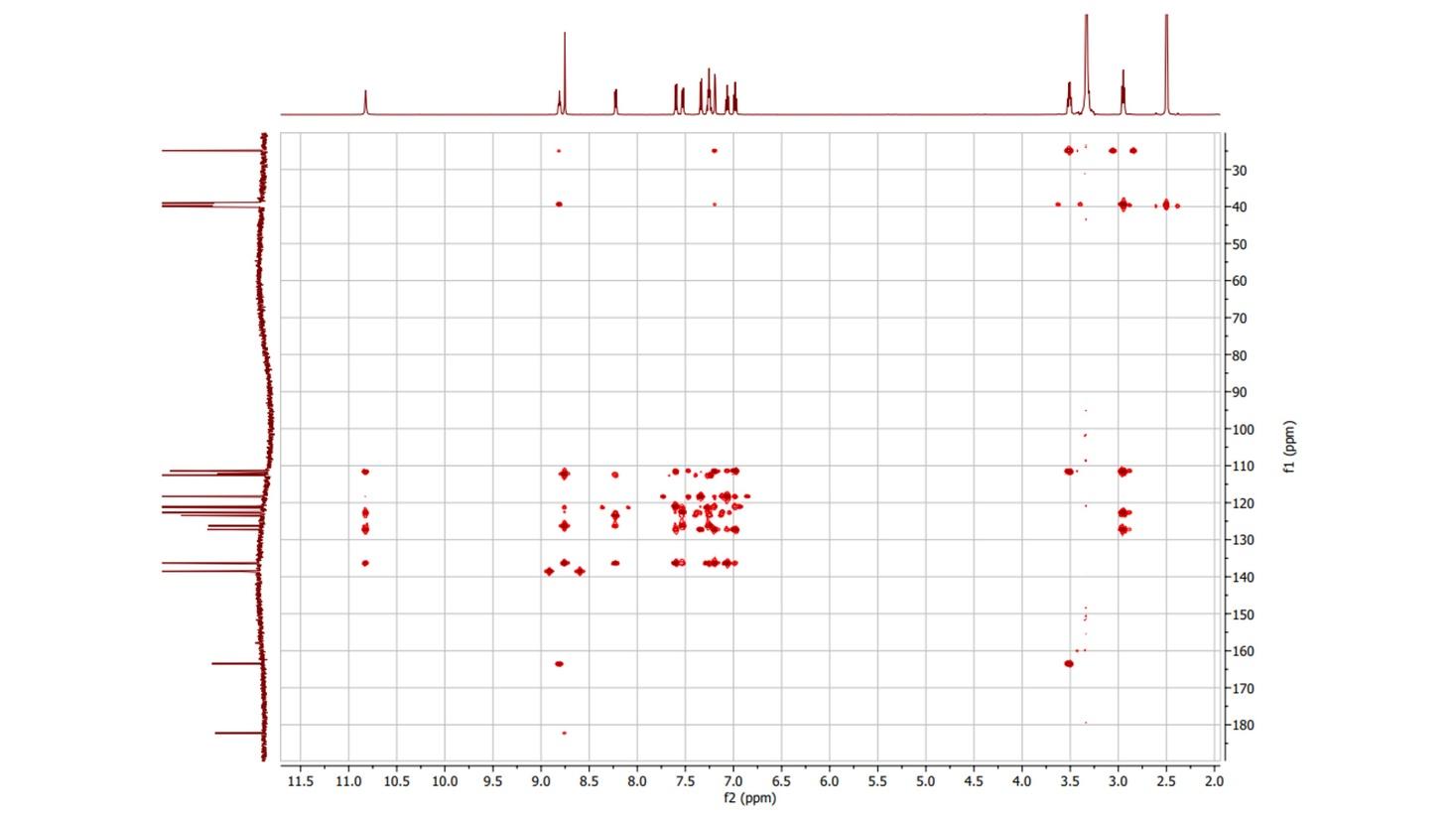


**Fig. S13.** HMBC spectrum of compound **1** (DMSO-*d*_6_, 600/150 MHz).


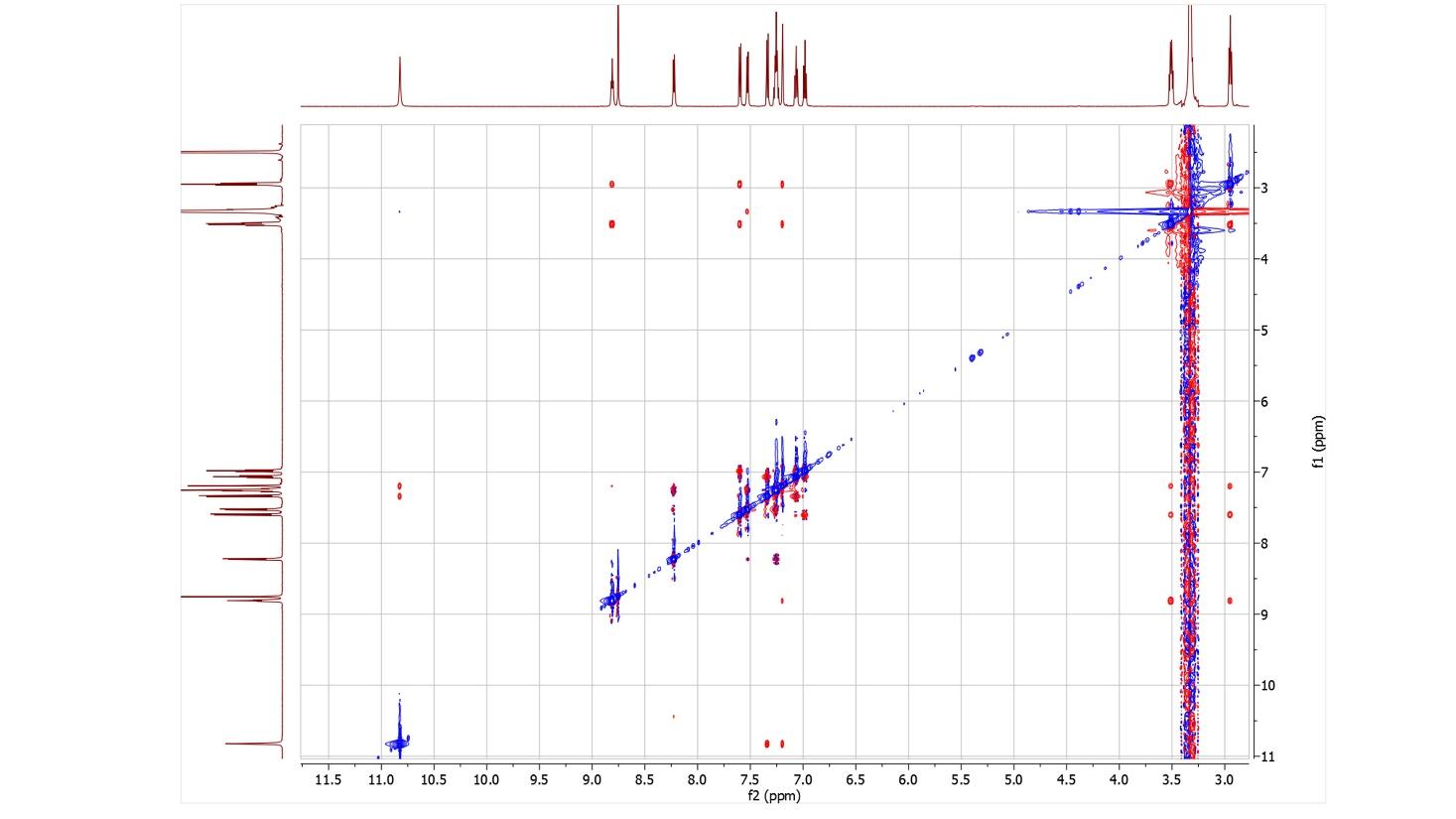


**Fig. S14.** NOESY spectrum of compound **1** (DMSO-*d*_6_, 600 MHz).


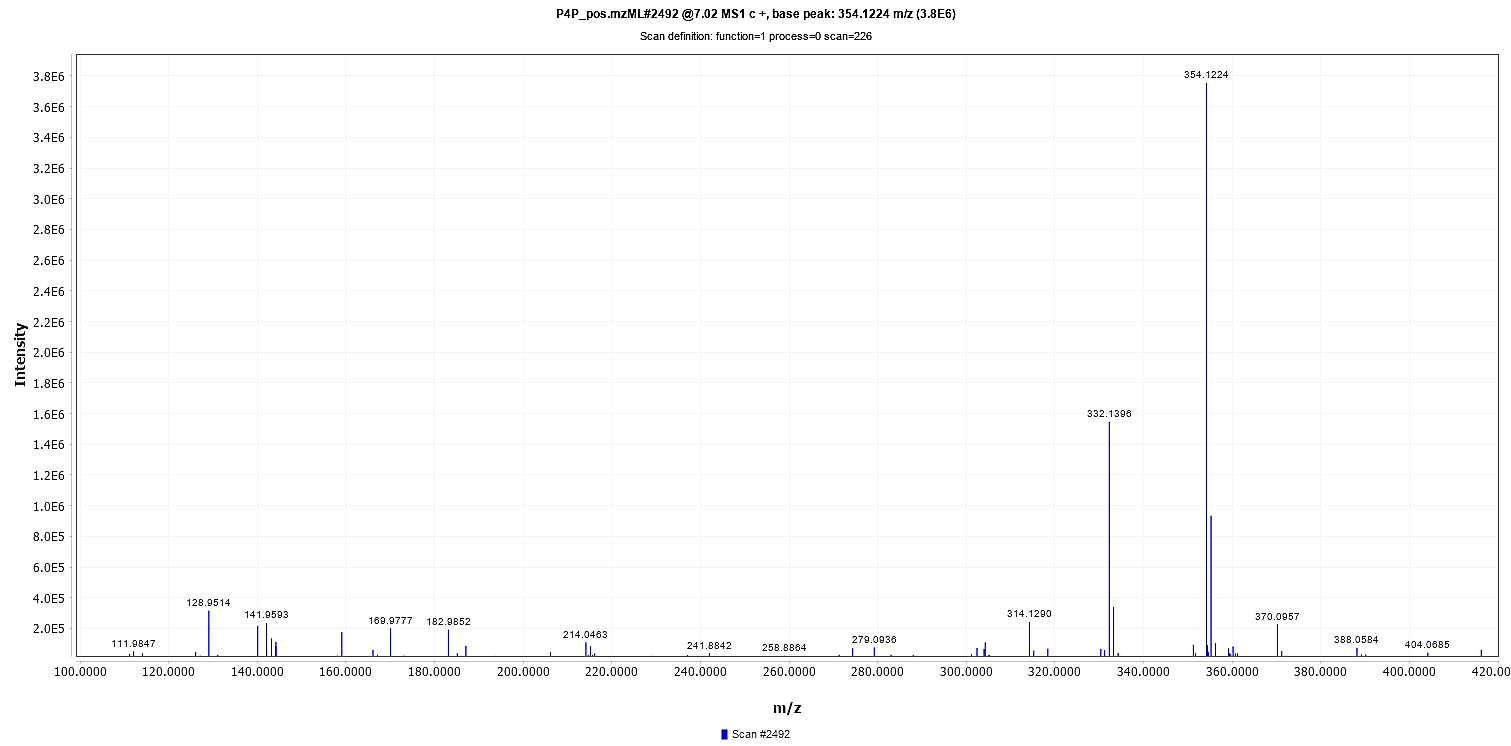


[M+Na]^+^

[M+H]^+^

**Fig. S15.** HR‐ESIMS(+) spectrum of compound **1**.


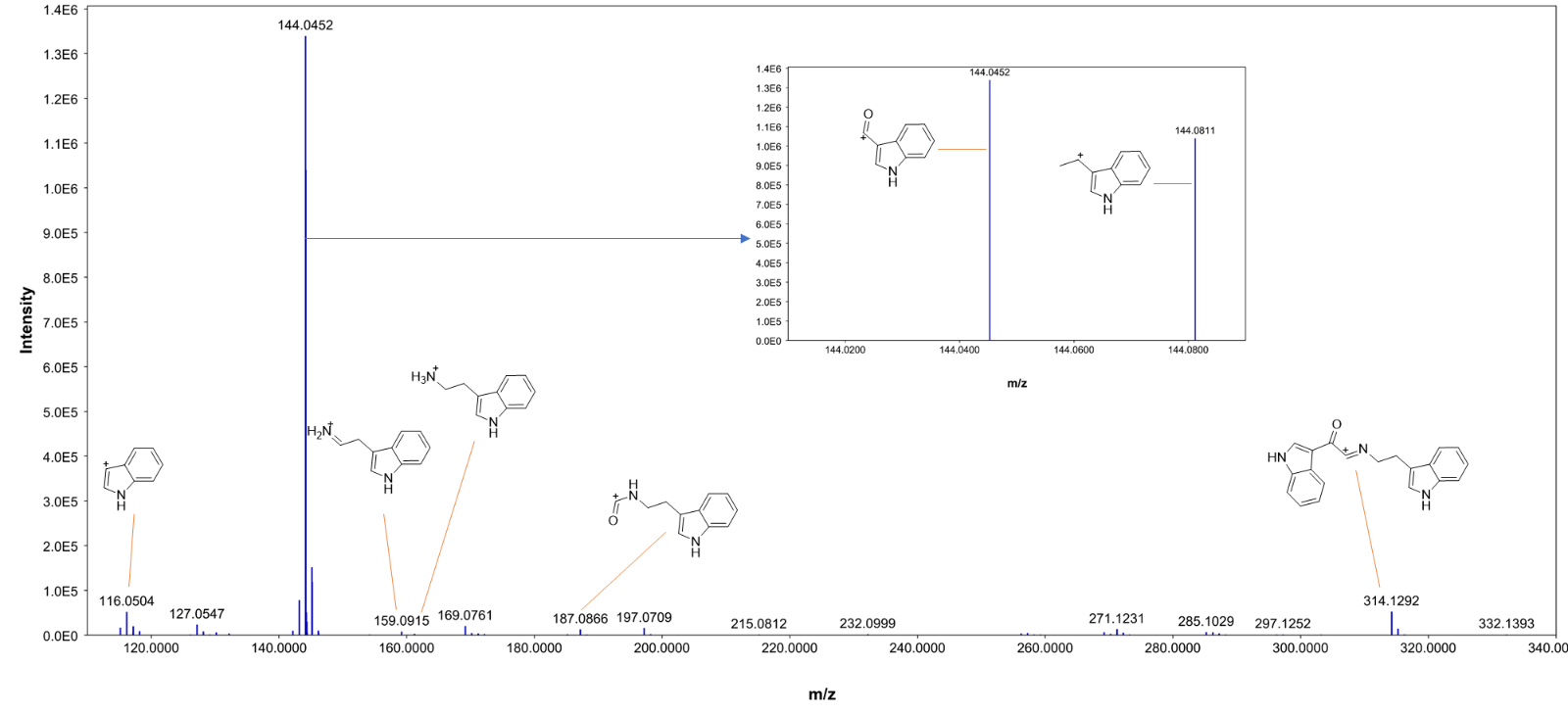


**Fig. S16.** HR‐ESIMS/MS spectrum of [M+H]^+^ ion of compound **1** with fragments annotation.

**Extended methods**

*Sampling from the deep-sea*

Marine sediments were collected from a previously unexplored region (GPS: -58.035554 -61.304726) in the SST, Antarctica, using equipment on board the EU funded EUROFLEETS2-2013, BIO Hesperides research vessel in conjunction with the PharmaDEEP (<https://www.eurofleets.eu/access/previous-calls/eurofleets2-regional-2-call-results/eurofleets2-funded-project-pharmadeep-results/>) project (2015) (Fig. 7). Samples were collected from a range of 1950.2-5100 m in water depth, and between 0-10 cm from the seabed at temperatures close to 0 °C. These samples were then couriered at a temperature of 4 °C and stored at -20 °C.

*Bacterial isolation from deep-sea sediments*

From the PharmaDEEP sediments, bacteria were isolated using culture-dependent techniques for the enrichment of marine microorganisms and cryopreserved in glycerol at -80 °C. Particularly, the solid media used included Marine broth (MB), Nutrient broth (NB), Artificial seawater (ASW) and Sulphate reducing broth (SRB) containing 10% (w/v) gellan gum as a solidifying agent. Serial dilutions were made of the respective sediments and spread-plated onto the media. After ≥8 weeks of incubation at 10 °C, in both aerobic and anaerobic conditions, 25 visible colonies were selected according to differences in morphology. Thereafter, the colonies were inoculated in their respective isolation (liquid) media and cultivated at 10 °C for 5-10 days in agitation, followed by preparation of cryopreserved glycerol stocks, which were stored at -80 °C.

*Genome sequencing, assembly, and annotation*

Isolate WUR7 was subjected to *de novo* WGS using Pacific Biosciences RS II single-molecule real-time (SMRT) technology. SMRT sequencing was followed by Illumina NextSeq™ 500 sequencing for error-correction of the *de novo* assembled genomes. Therefore, DNA was isolated using Qiagen Genomic-tip 100/G (Qiagen, Hilden Germany) according to the instructions of the manufacturer. SMRTbell™ template library was prepared according to the instructions from Pacific Biosciences, Menlo Park, CA, USA, following the Procedure & Checklist – Greater Than 10 kb Template Preparation. Briefly, for preparation of 15 kb libraries 8 µg genomic DNA from strain WUR7 was applied unsheared. DNA was end-repaired and ligated overnight to hairpin adapters applying components from the DNA/Polymerase Binding Kit P6 from Pacific Biosciences, Menlo Park, CA, USA. Reactions were carried out according to the manufacturer´s instructions. BluePippin™ Size-Selection to greater than 4 kb was performed according to the manufacturer´s instructions (Sage Science, Beverly, MA, USA). Conditions for annealing of sequencing primers and binding of polymerase to purified SMRTbell™ template were assessed with the Calculator in RS Remote, PacificBiosciences, Menlo Park, CA, USA. 1 SMRT cell was sequenced per strain on the PacBio RSII (PacificBiosciences, Menlo Park, CA, USA) taking one 240-minutes movies. Libraries for sequencing on Illumina platform were prepared applying Nextera XT DNA Library Preparation Kit (Illumina, San Diego, USA) with modifications according to Kishony et al.(2). Samples were sequenced on NextSeq™ 500. Genome assembly was performed applying the RS_HGAP_Assembly.3 protocol included in SMRT Portal version 2.3.0 applying a target genome size of 10 Mbp. The genome assembly revealed seven contigs summed up to a total genome size of 3.6 Mbp. Error-correction was performed by a mapping of the Illumina short reads onto finished genomes using the Burrows-Wheeler Aligner bwa 0.6.2 in paired-end (sample) mode using default setting (3) with subsequent variant and consensus calling using VarScan 2.3.6 (4). Automated genome annotation was carried out using Prokka (5). The genome has been deposited at NCBI GenBank under Accession number CP045067.

*Type strain genome server (TYGS) for genome-based taxonomy*

A complete genome-based taxonomic analysis was performed using the Type Strain Genome Server (TYGS), a free bioinformatics platform available under the URL <https://tygs.dsmz.de/> (6). In brief: **1)** an algorithm is used to extract the highest scoring 16S rRNA gene sequence from the query genome; **2)** a BLASTn sequence comparison is then conducted against all available type strains in the database; **3)** Genome BLAST distance phylogeny (GBDP) distances are calculated between all 16S sequence pairs with a high enough bitscore; **4)** distances are sorted and n number of type strain genomes, most closely related to the query genome are found; **5)** for previously determined subset of strains: all pairwise intergenomic sequences are calculated using the GBDP. The results include a **1)** genome-based phylogenetic tree; **2)** 16S rRNA gene tree; **3)** digital DNA-DNA hybridisation (DDH); **4)** affiliations to (sub-) species clusters and **5)** differences in G+C content.

*Phylogenetic inference*

The resulting intergenomic distances were used to infer a balanced minimum evolution tree with branch support via FASTME 2.1.4 including SPR postprocessing (7). Branch support was inferred from 100 pseudo-bootstrap replicates each. The trees were rooted at the midpoint and visualised with PhyD3 (8).

*Type-based species and subspecies clustering*

The type-based species clustering using a 70% dDDH radius around each of the 10 type strains was done as previously applied. The resulting groups are shown in Table S2. Subspecies clustering was done using a 79% dDDH threshold as previously introduced (9).

*Genome mining using antiSMASH*

The ‘antibiotics and secondary metabolite analysis shell—antiSMASH’ (https://antismash.secondarymetabolites.org) web server was used as a tool for identifying and analysing BGCs from the WUR7 (10). Prokka annotated genome sequence data was submitted to antiSMASH 5.0 by using default parameters and incorporation of the ClusterFinder algorithm.

Bioinformatic analysis

The 3-D structure of WUR7_ADC1 was determined by comparative analysis with the known 3-D structure of a homologous tryptophan decarboxylase ZP_02040762*.* This was achieved by doing a homology search of WUR7_ADC1 on PDB database. It was then modelled to the highest scoring match, ‘4OBV_A.pdb’, using UCSF Chimera and Modeller software packages; thereby creating three models. The quality of each model was assessed through PDBsum server (11). The whole Procheck structural analyses (12) were performed on all the models (data shown only for the best model), i.e. evaluation of conformations of residues compared to the allowed areas in the Ramachandran plot (Fig. S4 Ramachandran Plot). The model with higher stereochemical quality indices (G factors average= -0.02) was employed to create the images displayed by using the molecular graphics software VMD (13).

*Bacterial cultivation*

Small-scale cultures were prepared by inoculating 1 CFU into 3 mL of medium and incubating them at 20 °C with 160 rpm for 3 days. Thereafter, the pre-inoculum was used to inoculate a 500 mL flask with 100 mL of the same media at 0.01 OD 600/mL for 5 days at 20 °C, with agitation set at 180 rpm. Thereafter, to generate larger amounts of crude extract for purification, structural elucidation, and bioactivity assessments, the optimal culturing conditions were employed adopting the same protocol while increasing the volume up to 2.1 L.

*Metabolite extraction and SPE fractionation*

Spent culture media were centrifuged at 7500 x *g* at 4 °C for 45 minutes to separate the cells from the supernatant. Thereafter, the cell-free supernatant was subjected to repeated organic solvent extraction using 2 volumes of ethyl acetate (EtOAc) in a separating funnel, the organic phase was then dried using a rotary evaporator, therby generating the extracellular crude extract. This procedure yielded 650 mg of extracellular extract.

The crude extracellular extract obtained from the upscaled culture was subjected to fractionation by using Chromabond SPE C18 cartridges (5 g/45 mL) (Macherey-Nagel, Germany), which were selectively eluted with a gradient of MeOH in water. Five fractions were obtained as follows: 100% (v/v) H_2_O, 25% (v/v) MeOH/H_2_O, 50% (v/v) MeOH/H_2_O, 75% (v/v) MeOH/H_2_O, and 100% (v/v) MeOH. Each fraction was dried using the rotary evaporator and weighed.

*LC-MS analysis*

The routine LC-MS/MS profiling of small cultures – which produced WUR7 extracts - was operated on a Q-TRAP^TM^ 4500 (SCIEX, Framingham, MA, USA), in positive mode, connected to a Nexera X2 UHPLC (Shimadzu, Kyoto, Japan), equipped with a ACQUITY UPLC BEH 1.7 μm C18 column (2.1 x 50 mm).

The samples were dissolved in mass-grade MeOH, the mobile phase was composed by different ratio of phase A (100% H_2_O + 0.1% formic acid) and phase B (100% ACN + 0.1% formic acid) at a flow rate of 0.2 mL/min with the following gradient: initial 90% A – 10% B; 0 – 15 min, 0% A – 100% B; 15–20 min 0% A – 100% B. The MS was recorded in positive mode for ions in the mass range 150-1500 *m/z*, MS^2^ spectra were recorded for the most intense 5 ions mass in the range 150-1500 *m/z*, using a collision energy (CE): 40 eV, a scan rate: 10,000 Da/s, capillary voltage: 4.5 kV, source temperature: 200 °C, declustering potential:150 V.

UPLC-QToF-HRMS/MS analyses were carried out on an ACQUITY UPLC I-Class System coupled to the Xevo G2-XS QToF Mass Spectrometer (Waters®, Milford, MA, USA). The crude extracts were prepared at 0.01 mg/mL, and the Kupchan subextracts and SPE fractions were prepared at 0.001 mg/mL, with 1 µL injected into an Acquity UPLC HSS T3 column (High Strength Silica C18, 1.8 µm, 2.1 × 100 mm, Waters®) operating at 40 °C. A mobile phase system composed by different ratio of phase A: 0.1% formic acid in H_2_O and phase B: 0.1% formic acid in ACN was pumped at a flow rate of 0.6 mL/min, using the following linear gradient: initial, 99% A – 1% B; 0 – 11.5 min, 1% A – 99% B; 11.5–14.5 min 0% A – 100% B.

MS and MS/MS spectra, in positive mode, were recorded during the UPLC run with the following conditions: capillary voltage: 3.0 kV, sample cone voltage: 30 V, source temperature: 150 ^°^C. A scan range from 50 to 1200 Da was used, MS^2^ fragmentation was achieved with ramp collision energy: Low CE from 20–60 eV and a high CE of 40–80 eV. MS and MS^2^ data were acquired and analysed with MassLynx® software (Waters®, V4.1, Waters, Milford, MA, USA). All the solvents used in both analyses were MS-grade solvents.

*Molecular Networking*

The .raw files obtained were converted into .mzXML files by using the ProteoWizard tool MSConvert, in order to clean and to align them through MZmine (14) processing. Nodes arising from the methanol (Blank) and from the cultivation medium were deleted from all the samples. This workflow produced two files, one containing MS and MS/MS features (.mgf) and a quantification table (.csv). These files were submitted to the GNPS (15) server ([gnps.ucsd.edu](about:blank)) in order to build a Feature Based Molecular Network (FBMN) (16). The FBMN job was also reanalysed through the MolNetEnhancer workflow (17), aimed to a combined chemical classification of the cluster’s families, and Dereplicator Plus (18) was also run to improve the annotation of metabolites.

FBMN and MolNetEnhancer jobs are publicly accessible at the respective links: <https://gnps.ucsd.edu/ProteoSAFe/status.jsp?task=f52418c813d74418961f94d1d7304716>

https://gnps.ucsd.edu/ProteoSAFe/status.jsp?task=67d8a632d6f746bbbeb6b495ecaab82d.

For FBMN clusterisation, precursor ion mass-tolerance and the MS/MS fragment ion tolerance were set to 0.05 Da, furthermore, consensus spectra that contained less than 4 spectra were discarded. The network was created where the edges had a cosine score above 0.70, and 3 or more matched peaks. Edges further apart between two nodes were kept in the network if and only if each of the nodes appeared in each other’s respective top 10 most similar nodes. The maximum size of each cluster was set to 200 nodes. The spectra in the network were then searched against GNPS spectral libraries. All matches kept between network spectra and library spectra were required to have a score above 0.70 and at least 3 matched peaks. The clustered data were downloaded and imported into Cytoscape for visualisation.

*HPLC purification*

The initial HPLC separation was carried out using a 5 μm Nucleodur C_18_ reversed-phase HTec (250/10 mm) column connected to a Jasco HPLC equipped with a quaternary pump and a photodiode array detector. The mobile phase was composed of different ratio of Buffer A (H_2_O + 0.1% TFA) and Buffer B (ACN + 0.1% TFA) at a flow rate of 2.00 mL/min, employing the following gradient: initial 75% A – 25% B; 0 – 37 min, 15% A – 85% B; 37–40 min 0% A – 100% B. This way four fractions and three peaks were obtained, among them the most abundant peak P4 (5 mg) was further purified on the analytical column Phenomenex Luna 5 μm PFP column (250/4.6 mm). The following gradient: 75% A – 25% B; 0 – 10 min, 0% A – 100% B, with an operative constant flow of: 1 mL/min was used, to afford 1.5 mg of pure compound **1**.

*NMR characterisation*

Compound **1** was dissolved in 350 µL of DMSO-*d_6_* and transferred into a 5.0 mm Shigemi tube (SHIGEMI, Co., LTD., Tokyo, Japan). NMR spectra were recorded on a Bruker AV 600 spectrometer (600 and 150 MHz for ^1^H and ^13^C NMR, respectively, Bruker®, Billerica, MA, USA). The residual solvent signals for DMSO-*d_6_* (*δ*_H_ 2.50 and *δ*_C_ 39.51 ppm) were used as internal references. Data analyses were done with MestReNova (v.12, Mestrelab Research, Escondido, CA, USA).

*Description of compound 8,9-dihydrocoscinamide B (****1****)*

Compound **1**: 8,9-Dihydrocoscinamide B (*N-(2-(1H-Indol-3-yl)ethyl)-2-(1H-indol-3-yl)-2-oxoacetamide)* (**1**): white amorphous powder; ^1^H (600 MHz) and ^13^C (150 MHz) NMR data (Table S5); HR-ESIMS *m/z* 354.1219 [M+Na]+ (calcd for C_20_H_17_N_3_O_2_Na, 354.1219) (Fig. S15); HR-ESI-MS/MS (Fig. S16)

*Antimicrobial activity assessment*

The antimicrobial activity of 8,9-dihydrocoscinamide (**1**) was assessed against bacterial human pathogens *Staphylococcus aureus* DSM 346 as well as the *ESKAPE* panel. The latter included the gram-positive pathogens *Enterococcus faecium* DSM 20477, methicillin resistant *Staphylococcus aureus* DSM 18827 (MRSA), and the gram-negative bacteria *Klebsiella pneumoniae* DSM 30104, *Acinetobacter baumannii* DSM 30007, *Pseudomonas aeruginosa* DSM 1128 and *Escherichia coli* DSM 1576. All test strains were purchased from Leibniz Institute DSMZ (Braunschweig, Germany). The assay was performed in 96-well plates as previously described (19). Briefly, the compound was dissolved in DSMO and transferred in duplicates into a microplate for a final assay concentration of 200 µg/mL. The test organisms were cultivated overnight in a modified Tryptic Soy Broth (TSB) medium (1.2 % (w/v) TSB + 0.5 % (w/v) NaCl) except *E*. *faecium,* which was cultivated in M92 medium (3 % trypticase soy broth, 0.3 % yeast extract, pH 7.0-7.2). The pre-cultures were then diluted to an optical density (Abs_600 nm_) of 0.01 and 200 µL were added to each well of the microplate. After incubation for 5 h at 37 °C with 200 rpm 10 µL of a resazurin solution (0.3 mg ml-1 phosphate-buffered saline) was added to the microplates and after another incubation of 5 – 60 min at room temperature the fluorescence signal (560 nm/590 nm) was measured using the microplate reader (Tecan Infinite M200, Tecan, Männedorf, Switzerland). For *E. faecium*, the pH indicator bromocresol purple was used to determine the acidification caused by growing and the absorbance was measured (600 nm/ 690 nm reference). The resulting values were compared with a positive control (chloramphenicol for *S. aureu*s, *E. coli*, and *K. pneumoniae*, ampicillin for *E. faecium*, polymyxin B for *P. aeruginosa*, doxycycline for *A. baumannii*) and a negative control (DMSO) on the same plate. The percentage of inhibition was calculated by the rule of proportion. If the inhibition value was higher than 50% for the standard concentration of 200 µg/mL an IC_50_ determination was performed. For that, a dilution series was prepared and the IC_50_ value was calculated as the concentration that show 50% inhibition of viability based on a negative control (DMSO).

**SI References**

1. Bokesch HR, Pannell LK, McKee TC, & Boyd MR (2000) Coscinamides A, B and C, three new bis indole alkaloids from the marine sponge *Coscinoderma sp.* *Tetrahedron Letters* 41(33):6305-6308.

2. Baym M*, et al.* (2015) Inexpensive multiplexed library preparation for megabase-sized genomes. *PloS One* 10(5):e0128036.

3. Li H & Durbin R (2009) Fast and accurate short read alignment with Burrows–Wheeler transform. *Bioinformatics* 25(14):1754-1760.

4. Koboldt DC*, et al.* (2012) VarScan 2: somatic mutation and copy number alteration discovery in cancer by exome sequencing. *Genome Research* 22(3):568-576.

5. Seemann T (2014) Prokka: rapid prokaryotic genome annotation. *Bioinformatics* 30(14):2068-2069.

6. Meier-Kolthoff JP & Göker M (2019) TYGS is an automated high-throughput platform for state-of-the-art genome-based taxonomy. *Nature Communications* 10(1):2182.

7. Lefort V, Desper R, & Gascuel O (2015) FastME 2.0: A Comprehensive, Accurate, and Fast Distance-Based Phylogeny Inference Program. *Molecular Biology and Evolution* 32(10):2798-2800.

8. Kreft L, Botzki A, Coppens F, Vandepoele K, & Van Bel M (2017) PhyD3: a phylogenetic tree viewer with extended phyloXML support for functional genomics data visualization. *Bioinformatics* 33(18):2946-2947.

9. Meier-Kolthoff JP*, et al.* (2014) Complete genome sequence of DSM 30083(T), the type strain (U5/41(T)) of *Escherichia coli*, and a proposal for delineating subspecies in microbial taxonomy. *Standards in Genomic Sciences* 9:2.

10. Blin K*, et al.* (2019) antiSMASH 5.0: updates to the secondary metabolite genome mining pipeline. *Nucleic Acids Research* 47(W1):W81-w87.

11. Laskowski RA, Jabłońska J, Pravda L, Vařeková RS, & Thornton JM (2018) PDBsum: Structural summaries of PDB entries. *Protein Science : a publication of the Protein Society* 27(1):129-134.

12. Laskowski RA, MacArthur MW, Moss DS, & Thornton JM (1993) PROCHECK: a program to check the stereochemical quality of protein structures. *Journal of Applied Crystallography* 26(2):283-291.

13. Humphrey W, Dalke A, & Schulten K (1996) VMD: visual molecular dynamics. *Journal of Molecular Graphics* 14(1):33-38, 27-38.

14. Pluskal T, Castillo S, Villar-Briones A, & Oresic M (2010) MZmine 2: modular framework for processing, visualizing, and analyzing mass spectrometry-based molecular profile data. *BMC Bioinformatics* 11:395.

15. Wang M*, et al.* (2016) Sharing and community curation of mass spectrometry data with Global Natural Products Social Molecular Networking. *Nature Biotechnology* 34(8):828-837.

16. Nothias LF*, et al.* (2020) Feature-based molecular networking in the GNPS analysis environment. *Nature methods* 17(9):905-908.

17. Ernst M*, et al.* (2019) MolNetEnhancer: Enhanced Molecular Networks by Integrating Metabolome Mining and Annotation Tools. *Metabolites* 9(7).

18. Mohimani H*, et al.* (2018) Dereplication of microbial metabolites through database search of mass spectra. *Nature Communications* 9(1):4035.

19. Çiçek SS, Wenzel-Storjohann A, Girreser U, & Tasdemir D (2020) Biological Activities of Two Major Copaiba Diterpenoids and Their Semi-synthetic Derivatives. *Revista Brasileira de Farmacognosia : Orgao Oficial da Sociedade Brasileira de Farmacognosia* 30(1):18-27.
